# The Rho-Dependent Transcription Termination Is Involved in Broad-Spectrum Antibiotic Susceptibility in *Escherichia coli*

**DOI:** 10.3389/fmicb.2020.605305

**Published:** 2020-11-30

**Authors:** Md. Hafeezunnisa, Ranjan Sen

**Affiliations:** ^1^Laboratory of Transcription, Centre for DNA Fingerprinting and Diagnostics, Hyderabad, India; ^2^Graduate Studies, Manipal Academy of Higher Education, Manipal, India

**Keywords:** Rho, TolC efflux pump, *E. coli*, antibiotic susceptibility, transcription termination

## Abstract

One of the major ways of acquiring multidrug resistance in bacteria is via drug influx and efflux pathways. Here, we show that *E. coli* with compromised Rho-dependent transcription termination function has enhanced broad-spectrum antibiotic susceptibility, which arises from the inefficient TolC-efflux process and increased permeability of the membrane. The Rho mutants have altered morphology, distinct cell surface, and increased levels of lipopolysaccharide in their outer membrane, which might have rendered the TolC efflux pumps inefficient. These alterations are due to the upregulations of poly-N-acetyl-glucosamine and lipopolysaccharide synthesis operons because of inefficient Rho functions. The Rho mutants are capable of growing on various dipeptides and carbohydrate sources, unlike their WT counterpart. Dipeptides uptake arises from the upregulations of the di-peptide permease operon in these mutants. The metabolomics of the Rho mutants revealed the presence of a high level of novel metabolites. Accumulation of these metabolites in these Rho mutants might titrate out the TolC-efflux pumps, which could further reduce their efficiency. We conclude that the transcription termination factor, Rho, regulates the broad-spectrum antibiotic susceptibility of *E. coli* through multipartite pathways in a TolC-dependent manner. The involvement of Rho-dependent termination in multiple pathways and its association with antibiotic susceptibility should make Rho-inhibitors useful in the anti-bacterial treatment regimen.

## Introduction

The factor-dependent transcription termination process is well conserved among bacteria (D’Heygere et al., 2013; Ray-Soni et al., 2016). It is executed by the transcription terminator, Rho, a hexameric helicase having RNA-dependent ATPase activity (Banerjee et al., 2006; Mitra et al., 2017). Rho binds to the cytosine-rich unstructured sequences of nascent RNAs (the Rho utilization sites, “rut” sites) and due to its degenerate target sequences (Ciampi, 2006), transcription-termination of a large number of functionally unrelated operons is under its control (Cardinale et al., 2008; Peters et al., 2012). This has led to its involvement in various physiological pathways that are not connected. Some of the functions, where the Rho-dependent termination plays an important role, are maintenance of chromosome integrity, suppression of antisense and pervasive transcription, silencing of prophage and maintenance of its lysogeny, mediate sRNA and riboswitch action, prevention of replication and transcription collision, transcription and translation coupling, RNA remodeling, Mg^+2^ homeostasis and prevention of R-loops (Grylak-Mielnicka et al., 2016; Mitra et al., 2017).

Gram-negative bacteria are usually less susceptible to antibiotics than Gram-positive bacteria because they have an outer membrane with several drug efflux pumps embedded into it. The cell envelope of these bacteria is composed of an outer membrane (OM) and an inner membrane (IM) separated by a periplasmic space. The OM is an asymmetric lipid bilayer, in which phospholipids are exclusively partitioned in the inner leaflet and the lipid A moiety of lipopolysaccharides (LPS) forms the outer leaflet. IM is a phospholipid bilayer. The periplasmic space is primarily made of viscous peptidoglycans (Nikaido, 2003; Ruiz et al., 2006; Pages et al., 2008; Delcour, 2009). In addition to these physical barriers to the influx of antibiotics, multiple efflux pumps present in the cell envelope contribute further to reducing the antibiotic concentrations inside Gram-negative bacteria (Li et al., 2015).

Bacteria have evolved different efflux systems to tolerate a hostile environment. The efflux systems are required to secrete out many hazardous agents like bile salts, toxic metabolites, and heavy metals (Delmar et al., 2014). Multidrug resistance (MDR) or the polyspecific transporters constitute the major efflux systems. The MDR transporters are categorized into diverse super-families: ABC (ATP-binding Cassette), MF (Major Facilitator) and RND (Resistance-Nodulation-Cell Division). A few of the MDRs belong to smaller super-families: SMR (Small Multidrug Resistance) family [now it is part of the DMT (Drug/metabolite Transporter) superfamily] and MATE (Multidrug and Toxic Extrusion) family (Zgurskaya et al., 2015). The RND family of efflux pumps is clinically important because they are the major transporters that efflux out most of the antibiotics from both the periplasm and the cytoplasm, and hence, their presence renders Gram-negative bacteria less susceptible to antibiotics. This family of transporters is composed of three components: AcrA, AcrB, and TolC. The AcrB protein forms the inner membrane component and the TolC is the outer membrane pore, while the AcrA is the periplasmic adapter that connects both the AcrB and the TolC to form a continuous channel spanning both membranes (see Supplementary Figure S2C). This channel opens up in response to the binding of antibiotics (Wang et al., 2017). Gram-negative bacteria have been found to have all five superfamilies of efflux pumps, and this diversity contributes to their intrinsic resistance to diverse antimicrobials (Masi et al., 2017).

An elegant analysis of the antibiotic susceptibility profiling study in *E. coli* revealed that a varied range of genes is directly or indirectly involved in exhibiting various antibiotic sensitivities in addition to their specific target genes (Liu et al., 2010). Similarly, the metabolomic state and the complexity could also determine the nature of the antibiotic resistance in *E. coli* (Zampieri et al., 2017). We hypothesized that Rho protein being a pleiotropic regulator of gene expression, its inhibition, or mutations in its gene, could cause drastic changes in the *E. coli* physiology (Grylak-Mielnicka et al., 2016; Mitra et al., 2017), which is likely to influence the antibiotic susceptibility or resistance patterns of the bacteria. This hypothesis is also supported by an observation in our laboratory that the P1 transductants in the *rho* mutant background showed an unusual susceptibility to kanamycin compared to the WT strain.

Here, we show that *E. coli* strains carrying mutations in *rho* exhibited enhanced susceptibility to various antibiotics. These mutant strains exhibited defects in tolC efflux pump-dependent pumping out of the dye molecules and were synthetically defective with the *tolC* deletion under high antibiotic load. These strains have sticky cell surface textures decorated with a glycocalyx capsule and have an enhanced level of lipopolysaccharides, which could have altered the conformations of the TolC efflux pumps embedded in the OMs and affected their efficiencies. The metabolomics of the Rho mutants revealed that their cytoplasms contain metabolites at high concentrations. The Rho mutants were also able to utilize various unusual dipeptides and sugars. Excess metabolites in the cytoplasm of the Rho mutants might have titrated the TolC-efflux pumps, rendering them further less efficient in effluxing the antibiotics. We concluded that Rho-dependent termination is involved in the broad-spectrum antibiotic susceptibility of *E. coli* via a complex network of pathways in a TolC-dependent manner, which is a positive outcome of the pleiotropic nature of the Rho-function.

## Materials and Methods

### Materials

All the antibiotics except penicillin G were procured from Sigma, while penicillin G was from Ranbaxy. Ethidium Bromide (EtBr) was from Sigma. Dipeptides were from Bachem. Qiagen RNAeasy kit was used for RNA isolation. DNase I amplification grade, Superscript III-RT, RNase out, Ethylene diamine tetraacetic acid (EDTA), Dithiotreitol (DTT), and random hexamers were from Invitrogen. 25 mM MgCl_2_ and 10 mM dNTP solutions were from Thermo Scientific. Eva green RT-qPCR master-mix from GBiosciences was used for RT-qPCR. Sodium-deoxy cholate and Phenol were from USB, Sodium cacodylate was from Fluka. Lysine monochloride, Ruthenium Red, and Glutaraldehyde were from Sigma. Pancreatic DNaseI and RNaseA are from Sigma. Proteinase K and Silver Nitrate are from Emresco. Nile Red was from Thermo fisher scientific.

### Bacterial Strains

The bacterial strains used in this study (listed in Table 1) are derivatives of *E. coli* MG1655, while *E. coli* MC4100 was used in the phenotypic microarray analysis. RS1309 used to study the properties of the Rho mutants is a derivative of *E. coli* MG1655, in which chromosomal *rho* and *rac* prophage were deleted by P1 transduction. The kanamycin resistance gene that was transduced during the P1 transduction while bringing in the *rho:kan^*R*^* cassette was subsequently cured off. Since *rho* is an essential gene, the strain was provided with IPTG inducible, “shelter” plasmid pHYD1201 (*amp*^*R*^), expressing the WT Rho protein. The replication of the shelter plasmid is dependent on IPTG, and the *rho* is expressed from its own promoter. This plasmid could be removed if the strains are grown in the absence of Ampicillin and IPTG. The *rac* prophage was also deleted since it contains the *kil* whose expression causes lethality in the termination defective Rho mutants. Either WT *rho* or mutant *rho* (N340S and G324D) cloned in pCL1920 (spec^*R*^) was transformed into RS1309 followed by the shelter plasmid curing by growing in absence of the Ampicillin and IPTG. The removal of the shelter plasmid was routinely checked by streaking the strains on a LB-Ampicillin plate. Chromosomal deletions of different genes in RS1309 harboring pCL1920 WT *rho* or mutant *rho* were introduced by P1 transduction. The P1 lysates were prepared from the Keio collection carrying the deletion of the specific gene. Genes of the multidrug efflux system *acrAB-tolC* were deleted to generate RS1882, RS1883, RS1884 strains (Table 1), which were confirmed by PCR with a forward primer specific to the upstream region of the gene and the kanamycin specific reverse primer.

**TABLE 1:** Bacterial strains.

Strain No.	Description	References
RS1309	*E. coli* MG1655 Δ*rho* (marker less, Δ*rho:*FRT), Δ*rac (rac:Tet^*R*^)*, carrying shelter plasmid pHYD1201 (Amp^*R*^)	Shashni et al., 2014
RS336	MC4100 *galEp3*Δ*rho* with pHYD1201, trpE9851(Oc):Kan^*R*^ Amp^*R*^ Tet^*R*^	Chalissery et al., 2007
RS1882	RS1309 Δ*tolC* by P1 transduction:Kan^*R*^ Amp^*R*^	This study
RS1883	RS1309 Δ*acrA* by P1 transduction:Kan^*R*^ Amp^*R*^	This study
RS1884	RS1309 Δ*acrB* by P1 transduction:Kan^*R*^ Amp^*R*^	This study
RS1885	RS1309 Δ*uhpT* by P1 transduction:Kan^*R*^ Amp^*R*^	This study
RS2050-	RS1309 Δ*pgaA* made by P1 transduction	This study
**Plasmids**		
pHYD1201	*rho* subcloned from pHYD567 into *Hin*dIII-*Sal*I sites of pAM34 (pMB9; IPTG dependent replicon, Amp^*R*^)	This study
pRS317	pCL1920 cloned with *rho* (WT): spec^*R*^, strep^*R*^	This study
pRS725	pCL1920 cloned with *rho* (N340S): spec^*R*^, strep^*R*^	This study
pRS1106	pCL1920 cloned with *rho* (G324D): spec^*R*^, strep^*R*^	This study
pRS1109	pCL1920 cloned with *rho* (Y80C): spec^*R*^, strep^*R*^	This study
pRS342	pCL1920 cloned with *rho* G51V: spec^*R*^, strep^*R*^	This study
pRS397	pCL1920 cloned with *rho* Y274D: spec^*R*^, strep^*R*^	This study
pRS341	pCL1920 cloned with *rho* P279S: spec^*R*^, strep^*R*^	This study
pRS2043	pCA24N cloned with rfaH, cam^*R*^; from ASKA collection.	Kitagawa et al., 2005

### Growth Assays for Checking Antibiotic Susceptibility

The effect of antibiotics on WT Rho and mutant Rho strains were studied by allowing them to grow in different concentrations of antibiotic-containing LB medium. For growth assays, 198 μl aliquots of LB media with different concentrations of antibiotics were taken in triplicates in 96 well microtiter plates (corning). Bacterial cultures grown overnight in the presence of spectinomycin were used for subculture. 2 μl of the overnight culture was added into each well and growth profiles were derived by measuring absorbance at 600 nm at 37°C in a SpectramaxM5 Microtiter plate reader (Molecular Devices, United States). Standard errors were calculated from the three independent measurements. Optical densities were plotted against the time to generate the growth curves. To measure the kinetics of antibiotic effects (Figure 3), the RS1309 and its derivatives were grown in the same way as above in the microtiter plate, except that the required antibiotic was added at OD_600_ ∼0.3 and the growth was monitored for the indicated time. For the CFU assays to check the antibiotic susceptibility, all the strains were grown overnight in LB media at 37°C. The saturated bacterial cultures were serially diluted and 5 ml spots were applied onto the LB agar plates containing different concentrations of antibiotics and were further grown at 37°C for 16 h. The viable counts were measured from the lowest dilutions. The growth rates shown in Figures 2C–F were calculated from the linear portion of the slope of the semi-log plots following the methods described in Dhamdhere and Zgurskaya (2010).

### EtBr Efflux Assays

The efficiency of efflux systems in WT and mutant Rho strains was studied by EtBr efflux assays (Martins et al., 2013). 10 mg/ml stock solution of EtBr in Milli-Q water was prepared. Different concentrations of EtBr ranging from 0.5 to 5 μg/ml were added to the LB agar plates. WT Rho, mutant Rho and efflux deficient mutants, Δ*tolC*, Δ*acrA*, Δ*acrB* were streaked or suitable dilutions were spread or spotted on different EtBr plates (Figure 2A and Supplementary Figure S2A) and were allowed to grow for 12–14 h at 37°C. The EtBr fluorescence from the plates was observed in a UV trans-illuminator upon excitation at 365 nm.

### Fluorescence Spectroscopy to Measure EtBr Influx Kinetics

The time course of accumulations of EtBr in the MG1655Δ*tolC* strains (RS1882) expressing WT and the mutant Rho proteins were measured by monitoring the fluorescence changes of the dye. EtBr fluorescence enhances by many folds upon interaction with the nucleic acids. Overnight grown cultures of these strains were sub-cultured in LB media and grown until the OD_600_ reaches ∼ 0.4 (mid-log phase cells) and 2 ml of cells were harvested. The cell pellets were washed twice with minimal A media and were re-suspended in 900 μl of the same media (Glucose is not added to the media). Re-suspended cells were then transferred to a quartz cuvette. 100 μl of 80 μg/ml EtBr in buffer was added to give a final volume of 1 ml and the final concentration of 8 mg/ml of EtBr. The time course of fluorescence accumulation in WT Rho and mutant Rho strains was obtained in the time scan mode by exciting at 500 nm and measuring the emission at 585 nm in a Hitachi-7000 fluorimeter. The emission scan (550–650 nm) was also performed at different time intervals (15, 30, 60 min) with excitation at 500 nm.

### Nile Red Accumulation Assays

Nile red is a substrate to study RND type of efflux pumps since it accumulates in the periplasm. In the Nile red efflux assays (Spiekermann et al., 1999), different concentrations of Nile red containing plates (5 mg/ml and 10 mg/ml) were prepared. RS1309 derivatives expressing the WT and the mutant Rho proteins and the RS1882 (Δ*tolC*) strains were streaked in duplicates and were allowed to grow at 37°C for 15 h. Fluorescence was observed using a Zeiss lunar V12 stereomicroscope using a Rhodamine filter. To get Nile red-stained single-cell images, overnight cultures of RS1309 expressing WT and N340S and G324D Rho mutants and RS1882 (Δ*tolC*) strains were sub-cultured in 20 μg/ml Nile red containing LB media and were grown till mid-log phase. Cell pellets were then suspended in 100 μl of minimal media. The cells were fixed with 3.7% formaldehyde and incubated on ice for 15 min, following which cells were washed and re-suspended in minimal media and were observed under a confocal laser scanning microscope (LSM700, Carl Zeiss, Germany) using 63X objective and A555 nm laser (red channel).

### Effect of Antibiotic Load on Rho Mutants

The MG1655 strains, RS1882 and RS1309 (Δ*tolC* and WT) were transformed with pCL1920 plasmid expressing either WT or the mutant Rho proteins, following which the shelter plasmid carrying the WT *rho* was removed by growing the cells without IPTG. These strains (in triplicates) were grown overnight in LB media. The next morning the growth curves were followed in microtiter-plate growth assays in the presence or absence of the antibiotics, Chloramphenicol, and Gentamycin in the same way as described before.

### Assays to Measure the Adherence of the Cells to Microtiter Plate Wells

Overnight saturated cultures of the strains expressing WT and mutant Rho proteins were sub-cultured in 2 ml LB broth in the wells of the tissue culture plates and were allowed to grow for 4 h at 37°C in static condition. After 4 h, the media was discarded by gently tilting the plates. The wells were washed twice with LB media and the residual media was completely removed with a syringe. Bound cells were collected by re-suspending in 100 μl LB and serial dilutions of the suspensions were plated. The number of colonies formed was counted from the highest dilution and were multiple with the dilution values (10^–7^ or 10^–8^) were plotted. Errors were calculated from three independent-spotting assays. In Crystal Violet staining (0.5% in water) assays, bound cells in the wells were stained with 1 ml of 0.5% crystal violet for 15 min and washed twice with Milli Q water, air-dried for 30 min, and photographs were taken.

### Estimation of EPS on the Cell Envelopes of the Strains Expressing WT or the Rho Mutants

Extracellular polysaccharide (EPS) of these strains was isolated by the procedures described in Chris and Malcolm (1998) and Hancock and Poxton (1988), and the hexose moieties were colorimetrically estimated by the phenol-sulfuric acid method (DuBois et al., 1956). Overnight cultures were sub-cultured in 5 ml LB broth and allowed to grow till their OD_600_ reaches ∼0.4 at 37°C following which, the cell pellets were collected and were re-suspended in PBS with 0.5% of Formamide (2 ml PBS + 10 μl Formamide) and incubated for 10 min at room temperature with intermittent vortexing. Cells were then spun down and the supernatant was collected and 9 ml ice-cold acetone was added and incubated overnight at 4°C. EPS obtained as precipitate was collected by centrifugation at 4,000 rpm for 15 min followed by washing with 5 ml Acetone. Pellet was air-dried for 3–4 h at 37°C to remove the residual acetone. Pellet was dissolved in 1 ml Milli-Q water by heating at 42°C. For the estimation of sugar, 200 μl of 0.5% phenol was added to 400 μl of the sample in a glass test tube. One milliliter of concentrated H_2_SO_4_ was added and incubated for 30 min at 30°C. Absorbance was measured at 490 nm. The concentration of hexoses was obtained from a calibration curve with glucose.

### Estimation of LPS Contents

LPS was extracted by the hot phenol-water method as described in Rezania et al. (2011). The overnight cultures of RS1309 expressing either WT or the Rho mutants, N340S, and G324D were sub-cultured and allowed to grow until the mid-log phase (OD_600_ ∼0.4). 5 ml cells were harvested and the cell pellet was re-suspended in 200μl of 1X PBS containing 0.15 mM CaCl2 and 0.5 mM MgCl2 and sonicated for complete cell lysis. The cell lysate was treated with DNase I and RNase A for 1 h at 37°C and with proteinase K for 1–3 h at 60°C. Five microliter of 10 mg/ml DNase I, and RNase A and 10 μl of 10 mg/ml proteinase K were added. After the enzyme treatments, the lysate was vortex after the addition 4 μl of chloroform. To this equal volume of 90% hot phenol (65–68°C) was added and incubated at 65°C for 15 min with intermittent shaking. Samples were then spun at 11,000 rpm for 10 min and the upper aqueous layer was collected. 0.5 M sodium acetate was added followed by ethanol precipitation of the LPS. LPS pellets were obtained by spinning the samples at 8,000 rpm for 10 min at 4°C. Pellets were washed and re-suspended in water. Samples were mixed with 2X SDS-loading dye and were fractionated in 10% SDS-PAGE. The gels were silver stained to observe the LPS bands. In this method majority of protein and nucleic acids were removed from the samples.

### Growth Assays for Checking the Dipeptide Utilization

The dipeptides, Pro-Ala and Gly-Pro were procured from Bachem. W-salts minimal media (2X stock: K_2_HPO_4_ 4.2 g, KH_2_PO_4_ 1.8 g, dissolved in 200 ml Milli-Q water) with 1 mM MgSO_4_ was used in these studies. The indicated dipeptides were the sole nitrogen source. To check the dipeptide utilization efficiency of RS1309 strain expressing the WT and mutant Rho proteins, strains were grown in W-salts minimal media containing 0.2% glucose and one of the dipeptides (2.5 mM), and the growth was monitored by measuring the OD_600 *nm*_ in 30 min interval for 24 h at 37°C.

### Bile Salts Sensitivity Assay

The bile salts sensitivity (Thanassi et al., 1997) was assessed for the MG1655 strains expressing WT or Mutant Rho proteins by streaking the strains on the LB agar plates containing different concentrations of the bile salt, sodium deoxycholate (0.5–2%) and were allowed to grow at 37°C for overnight.

### Microarray Analyses

Microarray analyses were performed with mid-log phase cultures of RS1309 strains expressing WT and different Rho mutants essentially following the methods described in Kostakioti et al. (2013). RNA isolation and subsequent microarray analyses of these strains were performed by Genotypic Technology, Bangalore, India.

### RT-qPCR Analysis

MG1655 strains (RS1309) expressing either WT or mutant Rho proteins were sub-cultured from the overnight cultures. RNA was isolated from mid-log phase cultures using Qiagen’s RNAeasy kit following which residual genomic DNA in the RNA preparations was eliminated by DNase I treatment. Two microgram RNA was used for the synthesis of cDNA using Superscript III Reverse Transcriptase following the standard procedures. The amount of cDNA produced during the PCR cycles was monitored in real-time using SYBR green dye using the Applied Biosystems 7500 RT-PCR system. The threshold cycle Ct was calculated from the midpoint of the sigmoidal curve obtained by plotting the fluorescence intensity against the number of PCR cycles. 2^–ΔΔ*Ct*^ was used to calculate the fold change in the mRNA level for the mutants with respect to the WT, where Ct = the number of threshold cycle; ΔCt = Ct of target gene–Ct of internal control; ΔΔCt = ΔCt of mutant –ΔCt of WT. The level of *rpoC* mRNA was used as an internal control. Primer pairs were designed corresponding to the middle region of the test genes so that the PCR products should be ∼200 nt in size.

### Primary Metabolite Analysis

RS1309 expressing WT and N340S Rho mutants were grown until the mid-log phase (OD_600_ ∼0.4). At this stage, cells were harvested by spinning at 6,000 rpm. The resulting pellet was lyophilized in liquid nitrogen and sent for the analysis. Primary metabolite analysis of the log phase cells was carried out by West coast metabolomics center, UC, Davis^1^ using ALEX-CIS-GCTOF-MS technique (details of the methodology are found in Fiehn et al., 2008, 691–704)^2^. Mass spectral data were processed by Chroma TOF version 2.32 software. Deconvolution of mass spectra, baseline setup above the noise level, and peak detection at 5:1 signal/noise ratio were performed by this software. The generated spectral data was further processed by a filtering algorithm-rtx5 in the BinBase database. The spectra are matched to database entries and the unmatched spectra with signal/noise > 25 were given new database entries. Final quantification is reported as peak height (not as peak area) as it is more precise for low abundant metabolites^3^.

### Transmission Electron Microscopy (TEM)

The Bacterial strains RS1309 expressing either WT or N340S or G324D Rho proteins were grown until mid-log phase (OD_600_ ∼0.4). At this stage, 2 ml of cells were spun down and washed with 0.1 M sodium cacodylate buffer (pH7.2) and was re-suspended in 5 ml of 0.15% Lysine for either 1 or 4 h that was followed by the addition of 0.03% of Ruthenium Red and were incubated for 12–14 h (Fassel et al., 1998). After the staining, cells are washed twice with 0.1 M sodium cacodylate buffer (SCB) to remove the excess Lysine and Ruthenium red dye, and the cells were spun down. This procedure is optimized for proper visualization of the glycocalyx structure surrounding the cell membrane (Fassel et al., 1998; Erlandsen et al., 2004). The cell pellets were re-suspended in 100 μl of SCB. Five microliter cells were added over the copper grids and allowed to bind for 10 min and the excess cells were removed by soaking with Whatman filter paper by gently touching the copper grids from one edge. Images were obtained using the JEOL (model 2100) transmission electron microscope at 120 kV.

### Scanning Electron Microscopy (SEM)

The bacterial cells (RS1309 expressing WT and mutant Rho proteins) for SEM analysis were prepared in a similar way as described in TEM, except that the cells were fixed with 2% glutaraldehyde for 1 h at 4°C, following which cells were washed twice with 0.1M SCB. The cell pellets were then re-suspended in 100 μl of SCB. Then these cells were gradually dehydrated by suspending them in increasing concentrations of ethanol, following which they were centrifuged at 2,500 rpm for 10 min. Finally, the cell pellets were dissolved in 100% ethanol. Ten microliter of the sample was spread over lysine coated coverslips and air-dried for 30 min followed by gold sputtering. Images were taken in a Hitachi S-3400N scanning electron microscope operating at 5 kV.

### Confocal Microscopy

Mid-log phase cultures of RS1309 expressing WT and the Rho mutants were spun down and the pellets were resuspended in 1 ml of buffer 1X-PBS. Cells were fixed with Methanol/Paraformaldehyde prior to the DAPI staining. A drop (20 μl) of fixed cells were spread gently over lysine coated slides and were air dried for 5 min following which they were stained with 100 μl of DAPI (4,6-diamidino-2-phenylindole) in 1X-PBS (1:2,000 dilution) for 2 min in dark. Cells were then mounted with 20 μl of 80% glycerol and a coverslip was placed over it and borders were sealed. Imaging was performed using a confocal laser scanning microscope (LSM700, carl zeiss, Germany) using 63 × 1.4 NA objective and excitation was with a 405 nm laser. For measurements of the size of the cells, we measured 744 cells of N340S mutant, 500 cells of G324D mutant and 204 cells of the WT strain. WT cells are more homogenous, so less number of cells were measured 204. Zen 3.1 software was used to measure the lengths of the cells.

## Results

### Transcription Termination Defective Rho Mutants Are Susceptible to a Broad Spectrum of Antibiotics

**FIGURE 1:**
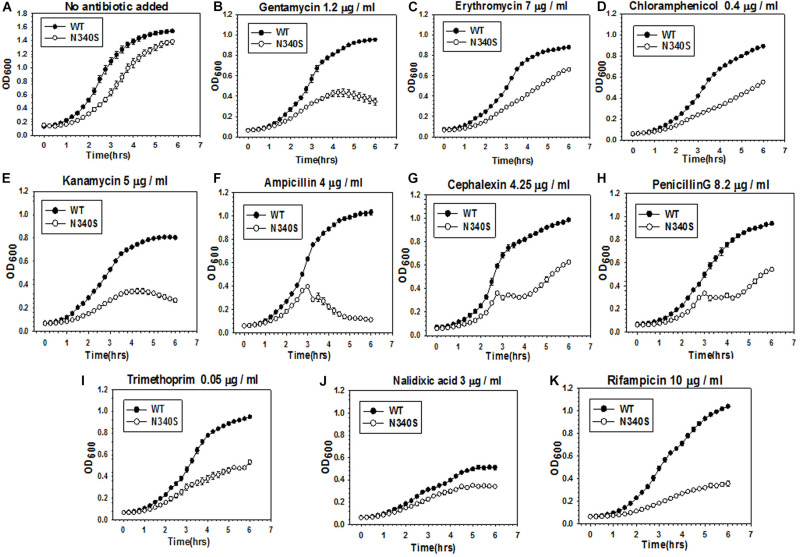
Comparison of antibiotic susceptibility of the *E. coli* MG1655 strains expressing the WT and the N340S Rho mutant. **(A–K)** Show the growth curves of the strain RS1309 expressing WT and N340S Rho in the presence of indicated types and amounts of antibiotics. Growth curves were obtained from the growth in the LB media supplemented with various antibiotics as indicated in a 96-well microtiter plate at 37°C. Error bars were obtained from at least three replicates in each case.

An observation in our laboratory that *E. coli* MG1655 strains expressing the Rho mutant, N340S, were more susceptible to Kanamycin (data not shown; please also see the growth curves in Figure 1 and Supplementary Figure S1), led us to check whether this susceptibility is specific one or mutations in *rho* cause broad-spectrum antibiotic susceptibility. We used the strain RS1309 with a chromosomal deletion in *rho*, in which a “shelter” plasmid (pHyd1201) carrying the WT *rho* was replaced with a low copy number plasmid, pCL1920, expressing either the WT or the mutant (N340S or G324D) Rho proteins. These two mutant Rho proteins are transcription termination-defective (Chalissery et al., 2007). We chose the following antibiotics for this study. Kanamycin and Gentamicin are aminoglycosides that specifically target the 30 s ribosomal subunit, Erythromycin is a macrolide and Chloramphenicol is an amphenicol, both acting on the 50 s ribosomal subunit. Ampicillin, Cephalexin, and Penicillin G are β-lactams that specifically act on the peptidoglycan layers of the cell wall. Trimethoprim inhibits dihydrofolate reductase. Nalidixic acid, a quinolone antibiotic, inhibits bacterial DNA Gyrase, and Rifampicin binds to and inhibits the β subunit of bacterial RNA polymerase. We grew these strains in the presence of optimum concentrations of various antibiotics, as indicated in Figure 1 and Supplementary Figures S1A,B. The optimum concentrations (below the IC_50_ values, concentration at which 50% survival was observed) of the antibiotics were identified as the concentrations that exerted a minimal effect on the growth of the WT strain. These concentrations were obtained from the estimates of IC_50_ of these antibiotics for the WT and the Rho mutant strains given in Supplementary Table S1. Compared to the WT Rho strain, both the Rho mutants exhibited enhanced susceptibility toward most of the antibiotics that were used. This was also corroborated by the reduced values of IC_5__0_s of various antibiotics for the Rho mutants. We assessed the enhanced susceptibility from the reduced rates and amplitudes of the growth curves (Figure 1 and Supplementary Figure S1A). The highest susceptibility was observed for Kanamycin, Ampicillin, Rifampicin, and Gentamycin. The Rho mutants were least susceptible to Nalidixic acid. A downward slope of the growth curves of the Rho mutants in the presence of Ampicillin indicated lysis of the cells, which could be due to impaired cell wall formation. This indicates that the defects in the Rho-dependent termination induce a broad-spectrum (wide range of cellular targets) antibiotic susceptibility. The enhanced antibiotic susceptibility of the Rho mutants observed in the growth curves was also reflected in the colony-forming unit (CFU) assays shown in Supplementary Figure S1B.

### TolC-Efflux Pumps Are Less Efficient in Rho Mutants

The broad-spectrum antibiotic susceptibility in Gram-negative bacteria could arise from increased permeability of the cell membrane to the antibiotics, and also from the decreased rates of their efflux from the cytoplasm. The TolC-dependent efflux pumps (RND-superfamily) are known to efflux most of the antibiotics that we used in the present study (aminoglycosides, macrolides, β-lactams, rifampicin; Rosenberg et al., 2000; Julio and Nikaido, 2005; Piddock, 2006; Nikaido and Pagès, 2012). Antibiotics like tetracycline and chloramphenicol were shown to be effluxed by RND-type pumps in other bacteria (Bornet et al., 2003). We measured the efficiency of the TolC-pumps to efflux out small molecules from the cells by using two dyes, Ethidium Bromide (EtBr; amphipathic) and Nile Red (lipophilic) that are bona fide substrates for the RND type efflux pumps (Blair and Piddock, 2016), such as AcrAB-TolC. EtBr enters the cell by facilitated diffusion through the porins (Paixão et al., 2009), and binds primarily to the nucleic acids that enhance its intrinsic fluorescence. Nile red binds to membrane phospholipids and accumulates in the periplasm (Blair and Piddock, 2016).

**FIGURE 2:**
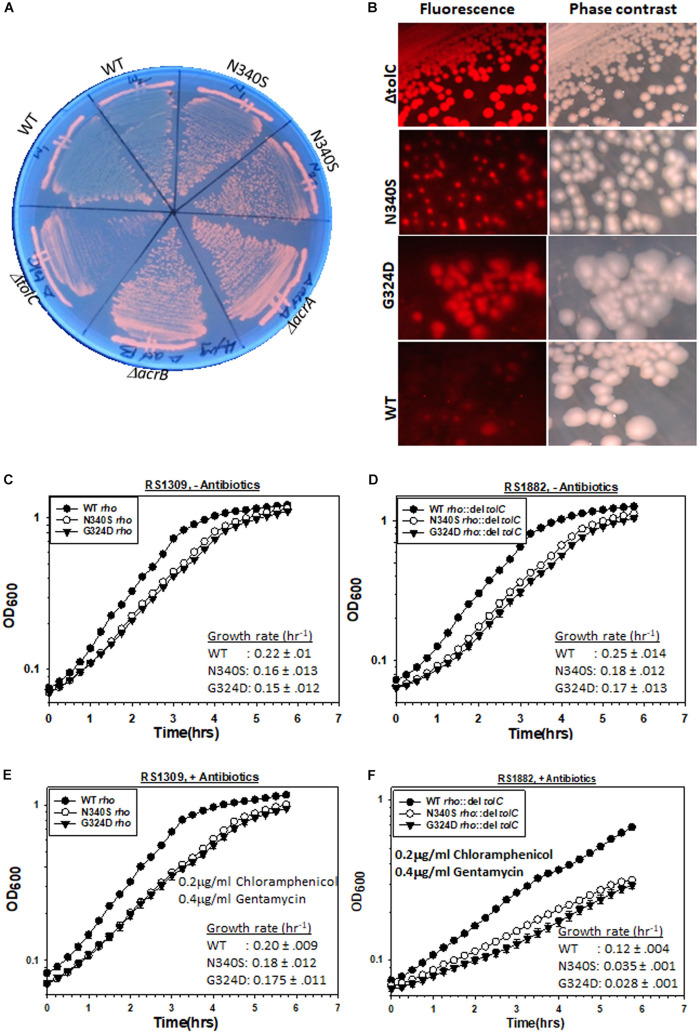
Measurements of the TolC-pump dependent efflux process by the MG1655 strain expressing WT or mutant Rho proteins. **(A)** Growth of RS1309 expressing the WT and the N340S Rho and the MG1655 strains having Δ*acrA* (RS1883), Δ*acrB* (RS1884), and Δ*tolC* (RS1882) on the LB plates containing 4 μg/ml Ethidium bromide. The plates were observed by UV-transilluminator set at 365 nm. The intense orange fluorescence in the N340S Rho and the efflux-pump defective mutants indicated the accumulation of EtBr. **(B)** The RS1309 derivatives same as above and the RS1882 were grown on LB plates containing 10 μg/ml Nile Red as indicated. The presence of the red fluorescence in the strains indicated defects in dye –efflux process. Cells were observed under a fluorescence microscope both in the fluorescence and phase contrast modes. **(C–F)** Growth curves of the RS1309 and RS1882 strains expressing WT and the Rho mutants in the absence **(C,D)** and presence **(E,F)** of the indicated antibiotics. The extent of survival was estimated from the OD values corresponding to the last time point. The reduction of survival of the RS882 strains is shown as vertical arrows relative to the level of RS1309 obtained from **(E)** (compare **E,F**). RS1882 is more susceptible to the antibiotic load in the presence of the Rho mutants, which indicates the essentiality of *tolC* in the Rho mutants.

**FIGURE 3:**
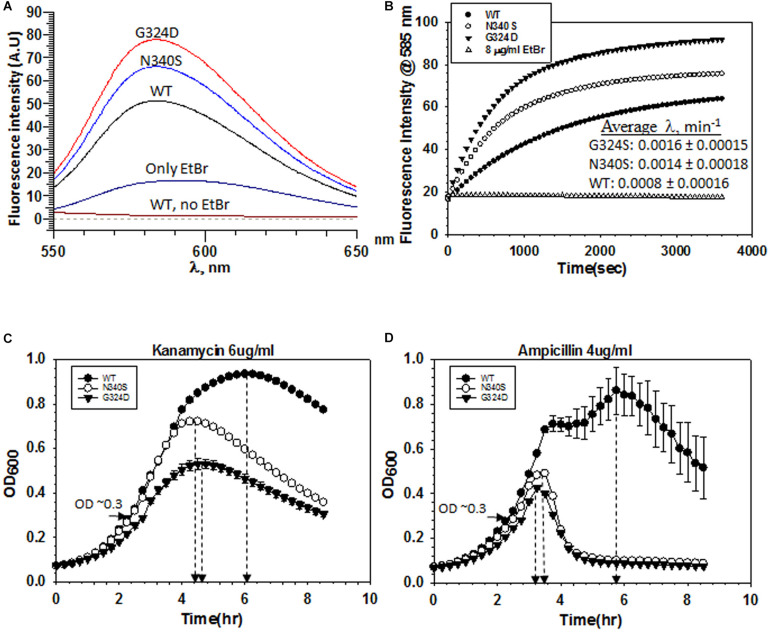
Measurements of influx kinetics. **(A)** Fluorescence spectra of EtBr upon addition of RS1309 (OD_600_ ∼ 0.4) expressing WT or the Rho mutants as indicated. The baseline was obtained by measuring the RS1309 suspension without the dye. EtBr without the strains gave residual fluorescence. **(B)** Time courses of the fluorescence enhancement of EtBr in the presence of RS1309 expressing the WT and the Rho mutants as indicated. The rate constants (λ) were measured from the curve-fittings using the exponential rise equation of the form: y = y_0_ [1–exp (–λt)]. Std error of means were calculated from two measurements. **(C,D)** Growth curves of the RS1309 strain expressing WT and the Rho mutants grown in microtiter plates. Indicated antibiotics were added to each strain at OD_600_ ∼ 0.3 as indicated by horizontal arrows. The time of onset of antibiotic action (the point of inflections of each curve) is shown by vertical arrows. Error bars were calculated from the measurements of three independent colonies.

The *E. coli* MG1655 strains (RS 1309) expressing WT or mutant Rho proteins were grown on LB-plates containing 4 μg/ml of EtBr for 12–14 h. As controls, MG1655 strains with deletions in the genes of the TolC-efflux pump components, *tolC, acrA*, and *acrB*, were also streaked alongside (Figure 2A). In these assays, the extent of staining of colonies was proportional to the EtBr accumulation inside the cells. We observed that the colonies were deep orange when the efflux pump genes were deleted or the Rho mutants were expressed (please see also the spotting as well spread-plate assays in Supplementary Figure S2A to compare the staining of each type of strain). This indicates an accumulation of EtBr inside these cells. Strains expressing WT Rho were lighter in color, which indicates less accumulation of the dye. Higher accumulation of EtBr in the Rho mutants was also evident from the enhanced fluorescence intensities obtained from fluorescence spectroscopy (see Figure 3A).

We performed similar assays with another dye, Nile red. RS1309 expressing either WT or mutant Rho proteins and RS1882 with a deletion in *tolC* were streaked on LB plates containing 10 μg/ml Nile Red, and were allowed to grow at 37°C for 15–16 h (Figure 2B). When *tolC* was deleted, all the colonies were uniformly red. The majority of the colonies of the strains expressing the Rho mutants were also found to be red and homogeneous (compare with their respective phase-contrast images), whereas the WT Rho-expressing strains were translucent and colorless. Enhanced Nile red accumulations in the Rho mutants and in the Δ*tolC* strain were also evident from the confocal microscopic images of a single cell (Supplementary Figure S2B). This indicates that the Nile Red accumulated in the strains with mutations in *rho* or deletion in *tolC*.

The above results suggest that like the Δ*tolC* strains, the strains carrying mutations in *rho* are likely to be defective in the TolC-dependent efflux process. The later strains may have a reduced level of expression of the TolC pumps. We measured the RNA expression level of *tolC* and the genes corresponding to the TolC-associated components of efflux pumps like AcrA, AcrB, MdtA, MdtE, and MdtF (Supplementary Figure S2D, left panel). The RT-qPCR PCR measurements of these RNAs revealed that the expression levels of *tolC* and *acrB* remained unchanged in the Rho mutants, whereas enhanced expression levels were observed for *acrA* and *mdt* genes (Supplementary Figure S2D, right panel). As the level of TolC-RNA remained the same, changes in the levels of some of its components should not change the number of efflux pumps in the Rho mutants compared to that of their WT counterparts. Therefore, it is likely that the observed defects in dye-efflux could be due to the defects in the function of TolC-efflux pumps and not due to the reduced expression levels of its components.

### Rho Mutants Are More tolC-Dependent Under High Antibiotic Loads

As the Rho mutants are defective in effluxing out the two dyes (Figures 2A,B), it is likely that compared to the WT strain, these mutant strains would become more dependent on the TolC-pumps in the presence of high antibiotic loads. We followed the growth curves of RS1309 (*tolC*^+^) and RS1882 (Δ*tolC*) strains expressing WT and the Rho mutants in the presence and absence of the two antibiotics, Chloramphenicol and Gentamycin (Figures 2C–F). These two antibiotics are bona fide substrates of the TolC-efflux pumps (Bornet et al., 2003). We observed the following. (I) In the absence of the antibiotics (compare Figures 2C,D), the deletion of *tolC* did not affect the growth rate of the WT or the Rho mutants significantly. (II) In the presence of the antibiotics (compare Figures 2E,F), deletion of *tolC* caused ∼1.7-fold (from 0.2 to 0.12 h^–1^ for the WT) and ∼5–6-fold (from 0.18 to 0.035 h^–1^ for the N340S and from 0.175 to 0.028 h^–1^ for the G324D mutant) reductions in the growth rate of the strains expressing WT and the Rho mutant proteins, respectively. This indicates that the growth of the Rho mutants under high antibiotic load is more dependent on the presence of TolC-efflux pumps compared to their WT counterparts. These effects were specifically dependent on the deletion of *tolC* because similar effects were not observed when an unrelated gene, *uhpt*, was deleted (Supplementary Figure S3A). These results further reinforce the proposition that the TolC-dependent efflux of antibiotics is less efficient in the strains expressing the Rho mutant proteins.

### Rho Mutants Have a Faster Rate of EtBr and Antibiotics Influx

Accumulation of dyes in the Rho mutants could also occur due to the higher influx-rates compared to their efflux rates. To measure the rate of dye-influx, we followed a time-dependent increase of the fluorescence of EtBr upon the addition of a Δ*tolC* strain (RS1882) expressing either WT or mutant Rho proteins using fluorescence spectroscopy. We used a Δ*tolC* strain to block the dye-efflux path so that we can measure only the influx kinetics. We measured the fluorescence intensity of EtBr at ∼585 nm (λ_*ex*_ at 500 nm), 30 min after the addition of the bacterial cells grown till the mid-log phase (OD_600_ ∼0.4) (Figure 3A). Among the three strains, the Rho mutants exhibited significantly higher fluorescence intensities of EtBr than what was observed in the presence of their WT counterparts, which indicated that more number of EtBr molecules have entered the mutant strains. Next, we followed the time-course of an increase in the fluorescence intensities (at 585 nm) of EtBr in the presence of different strains expressing WT or mutant Rho proteins (Figure 3B). The initial rates of the curves reflect the time taken by the dye molecules to enter the cells and bind the DNA. As the cell membrane poses a physical barrier for the entry of this dye, it can be assumed that the cell entry step(s) should limit the rate of the EtBr-DNA interaction. The rate constants would be dependent primarily on the entry step(s), which in turn is the measure of the permeability of the membranes to the dye. We observed that the composite rate constants (λ) of the EtBr influx-kinetics were twofold faster in case of the strains expressing the Rho mutants, which suggests that these mutants are more permeable to the dye (Figure 3B).

Next, we measured the kinetics of action of the two antibiotics, Kanamycin and Ampicillin, by following their effects on the growth curves of the RS1309 strains expressing either WT or the mutant Rho proteins. In these assays, the delay (lag time) in the onset of the growth inhibition by the antibiotics is proportional to the time taken by them to cross the cell membrane and reach their respective targets, and these steps are rate-limiting for the whole process of the growth inhibition. We added the indicated concentrations of the antibiotics (Figures 3C,D) when the cells reached OD_600_ ∼0.3 and continued to monitor the growth. We observed that the differences of lag time between the WT and the Rho mutant strains (time points at the onset of growth inhibition are indicated) were ∼1.5 and 2.5 h for Kanamycin and Ampicillin, respectively. These results suggested that these antibiotics took significantly less time to reach their respective targets in the Rho mutants, which could be interpreted as higher permeability of the cell membranes of these mutants to the antibiotics.

From the microarray profiles (Shashni et al., 2014), it is revealed that different types of membrane-bound transporters for sugars, amino acids, metal ions, etc. (Supplementary Table S2) are upregulated in the Rho mutants. The profiles did not show upregulation of specific antibiotic importers, ompC and ompF. So, most likely the higher antibiotic influx rates of the Rho mutants were due to the enhanced permeability (via passive diffusion) of the membranes toward the antibiotics. However, even though the levels of these two antibiotic-transporters were not enhanced, increased level of sugar and lipid moieties on the outer membrane (described in the later sections) could influence their permeability to the antibiotics.

The aforementioned results indicated that the mutant Rho expressing strains accumulate antibiotics because of the two reasons: (i) higher permeability of the membrane for the drugs and (ii) inefficient TolC-dependent drug-efflux process. The resultant effects of these two processes led to higher net influx rates of the drugs leading to their higher accumulation and made the strains more susceptible to antibiotics. The higher influx-rates of the antibiotics in the Rho mutants could be one of the reasons for the non-epistatic behavior of the Δ*tolC* strains expressing the Rho mutants.

The higher accumulation of the two dyes in the Rho mutants could arise because of the presence of a large number of dead cells with damaged cell membranes. We ruled out this possibility because these mutants exhibited similar sigmoidal growth curves (Figure 1A) and exhibited similar viable cell counts (Supplementary Figure S1B, no antibiotic plates) compared to their WT counterparts. These Rho mutants grow slowly compared to the WT (see Figure 1A and Supplementary Figure S1 for the growth curves and Supplementary Figure S1B for the colony size), due to which there are more undivided cells in the population of the mutants and they appeared as filamentous in Figure 6 (see below). Moreover, the kinetics assays of the dye- and the antibiotics-uptake (Figures 3B–D, 5A) were performed with the actively dividing mid-log phase cells.

We next explored the molecular bases of enhanced permeability of the membranes to the antibiotics and impaired functioning of the TolC efflux pumps in the Rho mutants.

### The Upregulation of PGA Operon in Rho Mutants Causes Bio-Film Like Formations in Log-Phase Cultures

**FIGURE 4:**
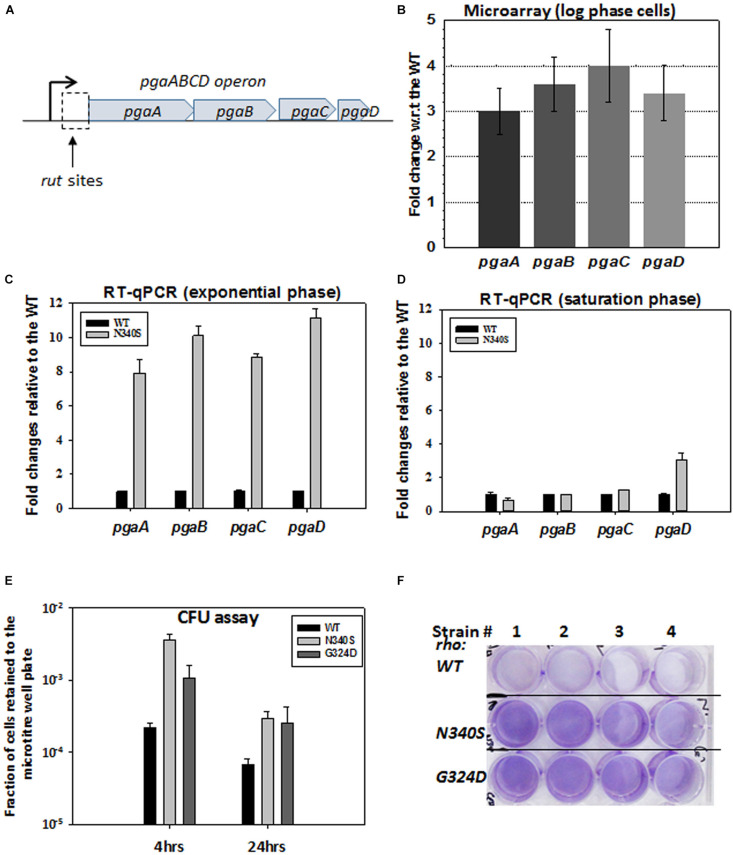
Rho mutant expressing cells form bio-film like structure during the log-phase growth. **(A)** The cartoon describes the *pga* operon. PgaA is an outer membrane porin that translocates dPNAG from the cytoplasm through the outer membrane. PgaB is an outer membrane lipoprotein involved in the deacetylation and hydrolysis of PGA. PgaC and PgaD forms inner membrane subunits of PGA synthase. **(B)** Microarray analyses showing the upregulation of the *pga* operon genes of the RS1309 strain expressing the N340S Rho mutant. **(C,D**) The RT-qPCR analyses of the *pga* operon genes of the RS1309 expressing the Rho mutant N340S. Fold changes were expressed relative to the WT strains. The RT-qPCR analyses of the strains grown until the mid-log phase **(C)** and those grown until the stationary phase **(D)** are shown separately. **(E)** shows the colony-forming unit (CFU) assays to study the adhesion properties of the different derivatives of the RS1309 to the bottom of the wells of a microtiter plate. The fraction of cells retained was calculated with respect to the unwashed plates for each of the strains. Each type of cell was allowed to grow in the microtiter plates in a static condition for the indicated duration. Retained cells were re-suspended in LB and spotted on LB agar plates. **(F)** Cells were grown until the mid-log phase similarly as in **(E**). Residual cells adhered to the bottom of the wells were stained with crystal violet and the excess dye was washed off. Extend of adherence of the cells to the bottom of the wells was indicated as the deep violet color of the wells in the Rho mutants.

The observed defects in the drug efflux function of the TolC efflux pumps or enhanced permeability to the drugs could be a direct consequence of the altered membrane architecture of the dividing cells expressing the Rho mutants. The microarray analysis of the strains expressing Rho mutants (Shashni et al., 2014) revealed that the *pgaABCD* operon (Figure 4A; see Supplementary Figure S3B for PGA organizations in the membrane) is highly upregulated in the strains expressing N340S Rho (Figure 4B). In an earlier study, a Rho-dependent terminator was shown to exist upstream of the *pgaA* gene of this operon (Figueroa-Bossi et al., 2014). Hence, the upregulation of this operon in the Rho mutants as observed in our microarray data was due to the presence of this Rho-dependent terminator. This operon expresses polyglucosamine, the polysaccharide moieties (β-1, 6-GlcNAc polysaccharide) of the biofilm structure of *E. coli* (Wang et al., 2004), and is expressed primarily during the stationary phase in the WT strain.

To confirm the microarray data, we performed RT-qPCR assays to measure the level of RNA expressed from the pgaABCD operon, during the log and the stationary phases (Figures 4C,D). Unlike the WT strain, all the genes of this operon were highly upregulated in the N340S Rho mutant during the log-phase growth, whereas, except *pgaD*, all the other genes were not significantly upregulated during the stationary phase growth. The RT-qPCR data were consistent with the microarray profile.

The expression of *pgaABCD* operon during the log phase is likely to produce the biofilm adhesion molecule, poly-β-1,6-N-acetyl-D-glucosamine (PGA), on the outer membrane of the Rho mutants. Hence, the Rho N340S mutant is likely to form a biofilm-like structure even during the log-phase growth. To test this hypothesis, we grew RS1309 expressing WT and N340S Rho in a static condition for 4 h (mid-log phase) and also for 24 h (stationary phase) in tissue culture plates. The presence of the polysaccharides of the biofilms allows the cells to adhere to the bottom of the wells of the tissue culture plates. Unbound cells were removed by washing, and the bound cells were re-suspended in fresh LB media, following which they were spotted on the LB-agar plates by serial dilution to measure the number of viable cells remained bound to the wells of the tissue culture plates. We observed that the numbers of cells of the mid-log phase cultures of the Rho mutant bound to the wells were 15–30-fold higher than its WT counterpart (Figure 4E). After 24 h, about fivefold more cells expressing the Rho mutants remained bound to the wells compared to the WT cells.

Crystal violet stains the bacterial cells bound to the wells in the static growth assays (Agladze et al., 2005; Merritt et al., 2005). In the same assays as described above, we observed an enhanced crystal violet staining of the wells, where Rho mutants were grown (Figure 4F). This further confirmed the enhanced adhering properties of the Rho mutants during the log-phase growth (Figure 4F). These data strongly suggest that the MG1655 strains expressing Rho mutant proteins have a higher level of PGA coating and have a biofilm-like structure during the dividing growth phase, which usually is not observed in the WT cells.

To further support the above results, we measured the level of extra-cellular polysaccharides (EPS) adhered to the cell envelope in the Rho mutants by colorimetry. We observed that in the log-phase, the strains expressing the Rho mutants have ∼3-fold more sugar moieties adhered to their cell surface (Supplementary Figure S3C), which is consistent with the existence of the biofilm-like structures on their cell surfaces during the log-phase.

### Upregulation of the Genes of Waa Gene-Family (*rfa* Operon) in the Rho Mutants Causes Alterations in the Outer Membrane

The aforementioned results indicated that the outer membrane architecture is significantly different in the strains expressing the Rho mutants. In *E. coli*, 19 *waa* (*rfa*) gene products are responsible for the formation of the lipopolysaccharide components of the outer membrane (EcoCyc database). The lipopolysaccharides embedded in the outer leaflet of the outer membrane has three components: (i) Lipid A, a phospholipid anchor, (ii) LPS core, a polysaccharide moiety, and (iii) O-antigen, contains oligosaccharide-repeating units (Raetz and Whitfield, 2002; Supplementary Figure S3B). *rfaH*, codes an antiterminator of the Rho-dependent termination, RfaH that enhances expression of several operons involved in the synthesis of lipopolysaccharides, exopolysaccharides, hemolysin, and sex factor by antitermination of Rho-dependent termination (Creeger et al., 1984; Bailey et al., 1992; Leeds and Welch, 1996; Artsimovitch and Landick, 2002). We reasoned that *rfaH* and the genes of the *waa* operon could be upregulated in the Rho mutants during the log-phase growth so that to form altered outer membrane or capsule formation in the log-phase, which in turn might affect the membrane permeability and the efficiency of the membrane-bound TolC-efflux pumps.

**FIGURE 5:**
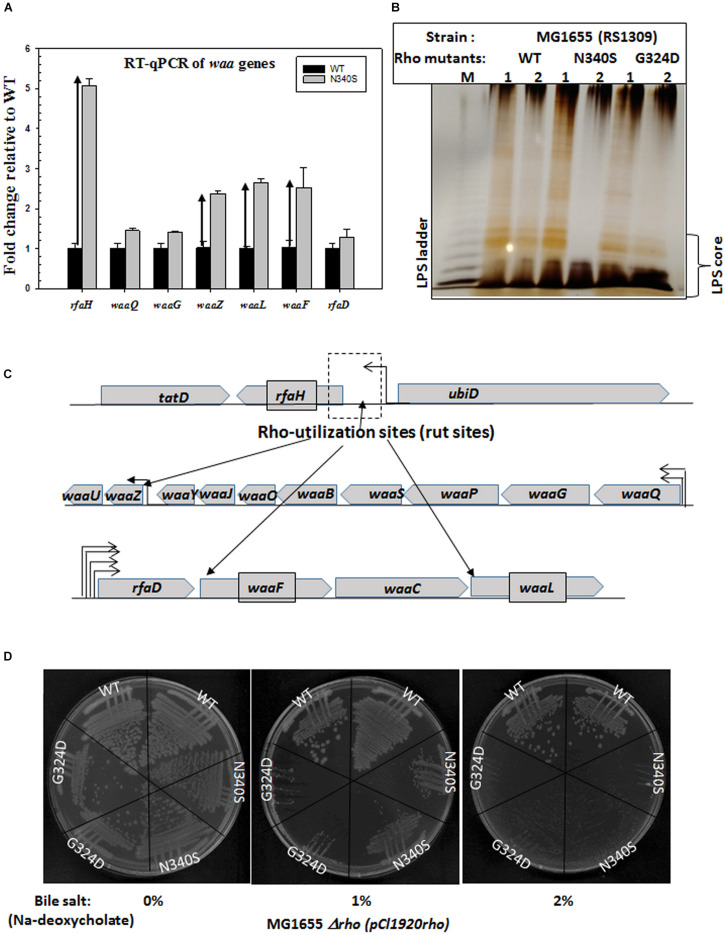
Upregulation of waa genes responsible for LPS synthesis and bile salt sensitivity**. (A)** Real-time expression analysis of *waa* genes by RT-qPCR. The fold increase in expression of the genes of the *waa* operon in the Rho mutant strains was measured relative to that of the WT strain. Upward arrows indicated the upregulation. **(B)** The silver-stained PAGE indicating the amounts of LPS in RS1309 strains expressing WT or the Rho mutants grown until the mid-log phase as indicated. The LPS bands are identified by comparing with the bands of commercially available *E. coli* (*E. coli* O55: B5) LPS (shown in lane M). **(C)** The operonic arrangement of different *waa* genes. Arrows indicated the putative positions of the Rho-dependent terminators (see Supplementary Figures S4A–C). **(D)** Bile salts sensitivity assays in the presence of different concentrations of Sodium-deoxycholate. RS1309 expressing WT and the Rho mutants were streaked on LB plates containing indicated amounts of the bile salt, Sodium-deoxycholate.

The upregulation of these genes was measured using RT-qPCR (Figure 5A). We have chosen *rfaH* and the following genes from the *waa* genes clusters: *waaQ* (heptosyl transferase 3), *waaG* (glycosyltransferase I), *rfaD* (ADP-L-glycero-D-mannoheptose-6-epimerase), *waaZ* (lps biosynthesis protein), *waaL* (O-antigen ligase), *waaf* (heptosyl transferase) (Figure 5C). We observed that along with the highly upregulated *rfaH, waaf*, *waaL*, and *waaZ* were also significantly upregulated in the Rho mutant N340S. Bioinformatics analyses revealed the presence of *rut* sites upstream of these upregulated genes (Supplementary Figures S4A–C). An earlier microarray study reported the upregulation of *waaA* and *waaL*, when the cells were treated with the Rho inhibitor, Bicyclomycin (Cardinale et al., 2008). The antiterminator, RfaH, interacts with the *ops* sequence elements of the DNA and modifies the elongating RNAP to overcome Rho-dependent terminators (Artsimovitch and Landick, 2002). The transcription units of the LPS synthesis genes (*waa* genes and *rfa* operon) have many *ops* elements and are reported to be under the control of this antiterminator (Belogurov et al., 2009). Hence, in addition to being controlled by the Rho-dependent termination, these are also under the regulation of RfaH-mediated antitermination. Therefore, the upregulation of the genes in the *waa* operon might have occurred both due to the RfaH action and also due to the impaired Rho-dependent termination in the N340S Rho mutants. These results suggest that the LPS content of the strains expressing the Rho mutants could be significantly higher than their WT counterpart.

Next, to support the RT-qPCR data, we directly measured the LPS contents of the WT and the Rho mutants (RS1309) grown until the mid-log phase (Figure 5B). The membrane fractions of the WT and the Rho mutant strains were extracted by hot-phenol methods (see the experimental procedure) and were analyzed by silver staining on an SDS-PAGE. We observed that both the Rho mutants have significantly higher LPS content during the log phase growth that is consistent with the elevated gene-expression levels of the *waa* family genes as shown in Figure 5A. The elevated LPS content is likely to alter the membrane fluidity, which could affect the conformational flexibilities as well as the efficiencies of the membrane-bound TolC-efflux pumps.

### Bile Salt Sensitivities of the Rho Mutants

Bile acids (or salts) are surface-active, amphipathic molecules, and their detergent activity damages cell membranes, and thereby cause growth defects in bacteria. Changes in the lipopolysaccharide (LPS), hydrophobicity, lipid fluidity, and fatty acid composition of the bacterial membrane can alter the sensitivity pattern toward the bile salts (Urdaneta and Casadesus, 2017). Hence, we used the bile salt sensitivity assay to probe the alteration in the membrane architecture.

We have grown the cells (RS1309) either expressing WT or mutant Rho proteins in the presence of varied concentrations of one of the bile salts, Sodium deoxycholate (Figure 5D). We observed that both the mutant Rho protein-expressing cells were significantly more sensitive to this bile salt compared to their WT counterparts. This further reinforces the proposition that the presence of the Rho mutants caused altered outer membrane conformation.

### Altered Cell Surface Features of the Rho Mutants

All the aforementioned results are strong indicators of an altered membrane as well as cell surface architecture caused by the defective Rho-dependent termination process. To further get direct evidence of these altered cell surfaces, we employed confocal and electron microscopy to visualize the WT and the Rho mutant cells.

**FIGURE 6:**
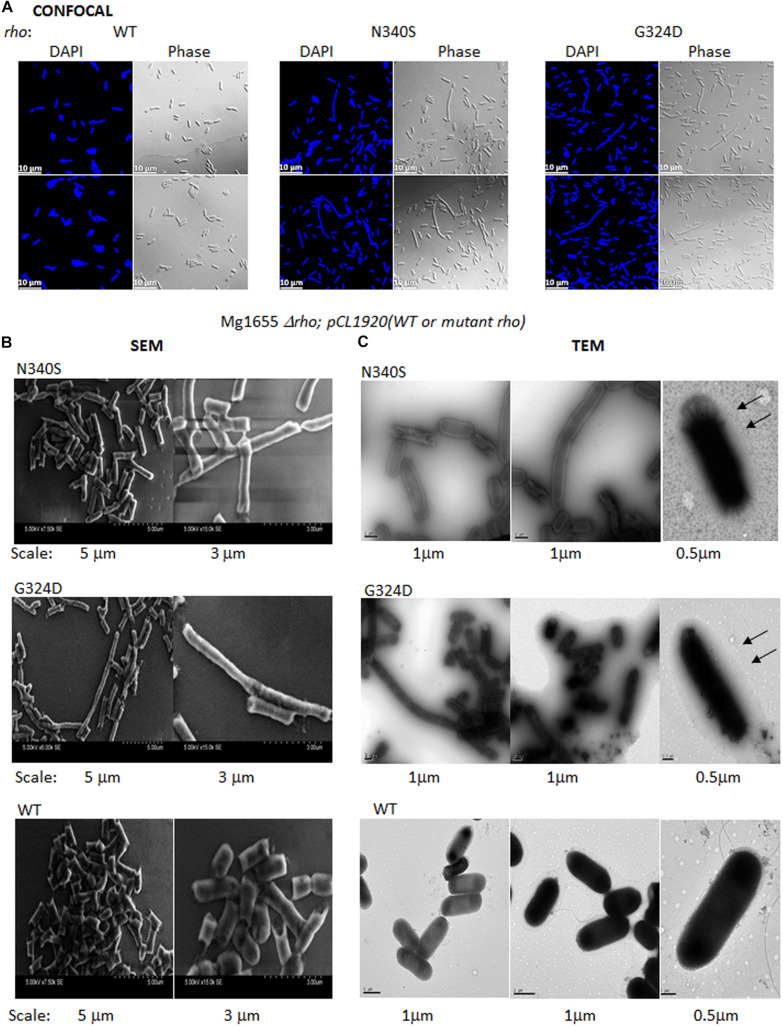
Confocal and electron microscopy. **(A)** Confocal microscopic analysis of RS1309 expressing WT and the mutant Rho proteins. The strains were stained with DAPI or visualized under phase contrast mode. The scales are indicated in the pictures. **(B)** Scanning electron microscopy (SEM) of the above the strains. Images with two magnifications are shown. The scales are embedded in the images. **(C)** Transmission electron microscopy (TEM) of the above strains stained by Ruthenium red. The magnifications and scale bars are indicated. The arrows indicated the presence of a glycocalyx layer outside the outer membrane specifically for the Rho mutants.

Figure 6A depicts confocal microscopic images of RS1309 strain expressing WT and the Rho mutants stained by DAPI (stains DNA) and their corresponding phase-contrast images. The WT Rho expressing strain showed homogenous rod-shaped cells with an average length of ∼3 μm. The cell length of the Rho mutants was more heterogeneous. Significant proportions (∼15%) were more than 3-fold longer (5–10 μm) than the WT strain. The longest cells observed in these mutants were > 20 μm. These elongated or filamentous cells arose from the slow or impaired cell division that could also alter the cell surface of these cells during the dividing phase.

To get a more detailed picture of the cell architecture and morphology, we visualized single cells from the mid-log phase culture using scanning (SEM) and transmission (TEM) electron microscopy. The SEM images revealed that the cells expressing the Rho mutants (RS1309 derivatives) have unusually long and tubular structures with a rough surface, those were in contrast to the small rod-shaped structure with a smooth surface observed for the WT cells (Figure 6B). The TEM images of the Ruthenium red-stained cells showed a distinct glycocalyx formation on the surface of the outer membrane (Figure 6C; indicated by arrows) of the cells expressing the Rho mutants. This glycocalyx deposition was not very prominent in the case of the WT cells. The presence of extensive glycocalyx on the surface of the Rho mutants could be correlated with the accumulation of poly N-acetyl-glucosamine and a high level of LPS moieties on the cell surface of these mutants during their log-phase growth.

The accumulation of poly N-acetyl glucosamine (PGA) in log-phase, a higher level of lipopolysaccharides in the outer membrane, and altered surface architecture could affect the conformations of the membrane-embedded TolC efflux-pumps as well as the membrane-permeability of the antibiotics. However, more direct assays to probe the altered conformations of the efflux pumps in the Rho mutants would be required to know the exact nature of these conformational changes *vis a vis* their reduced efficiencies.

### Accumulation of Unusual Metabolites in the Rho Mutants

The TolC-pumps effluxes out many small molecules as well as many metabolites that are toxic to the cells (Delmar et al., 2014). The changes in the physiology of *E. coli* caused due to the compromised Rho-dependent termination could accumulate unusual metabolites as by-products of the activation of new metabolic pathways (Supplementary Figure S5). These metabolites would require to be effluxed out by the TolC pumps, and this process could compete with the antibiotics efflux process by the same pumps. To test this hypothesis, we measured the ability to utilize different nutrient sources by the WT and the Rho mutants, and also measured the composition of the metabolites in these strains.

**FIGURE 7:**
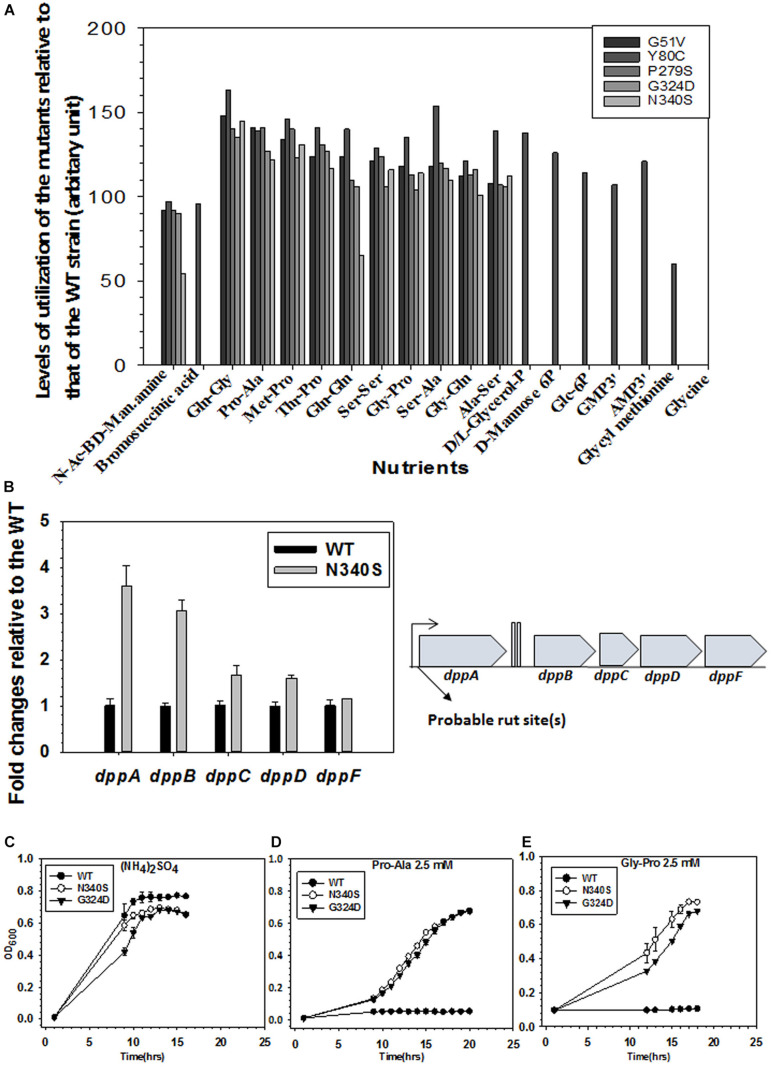
Metabolomics analyses of WT and the Rho mutants. **(A)** The phenotypic microarray analysis using the BIOLOG plates. Relative utilization of different nutrients as Carbon and Nitrogen sources by the different Rho mutants with respect to that of the WT strain (see also Supplementary Figure S6). The nutrients that have relative utilization above the value 50 (calculated from the area under the growth curves as shown in Supplementary Figure S6A) are plotted. **(B)** RT-qPCR analyses of the gene expressions of different gens of the *dpp* (dipeptide permease) operon. Probable Rho-dependent terminators at the beginning of the operon are indicated (see also Supplementary Figure S5). **(C–E)** Growth assays of RS1309 expressing the WT and the Rho mutants in minimal media supplemented with 2.5 mM of each of the Nitrogen sources as indicated Growth of three independent colonies were measured to obtain the error bars.

We generated phenotypic microarray (service provided by Biolog Inc., United States) profiles of the *E. coli* MC4100 strains expressing the WT and the various Rho mutants (Supplementary Figure S6A). In these assays, bacteria were grown in the presence of various Carbon, Nitrogen, Phosphorous, and Sulfur sources, and their respiration is measured in a time-dependent manner^4^. The ability or lack of ability of the different Rho mutants, relative to their WT counterparts, to utilize various nutrients indicates the up- or down-regulation of the various metabolic pathways. The respiration measurements were performed in the presence of 200 Carbon, 400 Nitrogen, 100 each of Phosphorous and Sulfur sources. The data revealed that the Rho mutants gained properties to utilize many new sugars, dipeptides, and nucleotides that could not be utilized by the WT strain (Figure 7A and Supplementary Figure S6B). The N340S Rho utilized 7 new carbon and 59 new nitrogen sources and lost the function of utilizing Glycine as a nutrient. The G324D Rho gained the ability to utilize 6 new carbon and 43 new nitrogen sources, whereas the Y80C Rho was able to utilize 13 new carbon, 204 new nitrogen, 17 new phosphorous, and 1 new sulfur sources. These data are consistent with the upregulation of many metabolic pathways in the Rho mutants as revealed from the detailed analyses of our earlier published micro-array profiles of these mutants (Supplementary Figure S5 and see Shashni et al., 2014).

As the Rho mutants gained the ability to utilize different dipeptides among many other unusual nutrients, we reasoned that the *dpp* (dipeptide permease) operon coding for the ABC transporter of the dipeptides could be upregulated in the strains expressing the Rho mutant proteins. The RT-qPCR data (Figure 7B) revealed that the expressions of the *dppA* and *dppB* of this operon are significantly upregulated in the N340S Rho relative to its WT counterpart. Bioinformatics analyses predicted the presence of *rut* sites in the untranslated region preceding the *dppA* (Supplementary Figure S7). Hence, we concluded that the *dpp* operon is under the control of Rho-dependent termination and its expression is significantly suppressed when a functional *rho* is present in the strain, and this dipeptide transporter is present in the cell membrane only when this termination process is compromised.

To further validate the aforementioned observations of gain of function phenotypes of utilizing dipeptides by the Rho mutants, we tested the ability of RS1309 strain expressing WT or Rho mutants to grow in the liquid minimal media supplemented with either Pro-Ala or Gly-Pro dipeptides. In these growth assays, the WT *E. coli* failed to grow, whereas the Rho mutants efficiently utilized the dipeptides and grew comfortably (Figures 7D,E). As all the three strains grew in the presence of the nitrogen source (NH_4_)_2_SO_4_, indicating that the utilization of the dipeptides by the Rho mutants were dipeptide permease transporter-dependent (Figure 7C).

The ability to utilize unusual nutrients by the Rho mutants lead us to hypothesize that these strains would accumulate many complex metabolites (as end-products of these new metabolic processes) in their cytoplasm compared to their WT counterparts, and they would also require to be effluxed out efficiently through the TolC efflux pumps. To measure the nature of metabolites in the strains expressing WT and mutant Rho proteins (RS1309), we performed primary metabolome analyses of these strains using GC-MS/TOF. The nature and level of the metabolites obtained from the Rho mutants were compared to those obtained from the WT strain (Supplementary Figures S8, S9). The physiological levels for 703 metabolites were measured, of which 185 metabolites could be characterized and 518 were uncharacterized. Seventy one and seventy seven metabolites were upregulated in the Rho mutants, N340S, and G324D, respectively. Consistent with these analyses, we also observed from the micro-array profiles (Shashni et al., 2014) that many different transporters of small molecules are upregulated in the Rho mutants (Supplementary Table S2).

This metabolomics analysis is consistent with our hypothesis of the accumulation of higher levels of unusual metabolites in the cytoplasm of the Rho mutants. These metabolites could be toxic to the cells, if not efficiently effluxed out using the TolC pumps (Rosner and Martin, 2009; Delmar et al., 2014). This higher level of complex metabolites is likely to “titrate” the TolC-efflux pumps by competing out the antibiotics, which might further contribute to the reduction in net efflux rates of the antibiotics.

## Discussion

**FIGURE 8:**
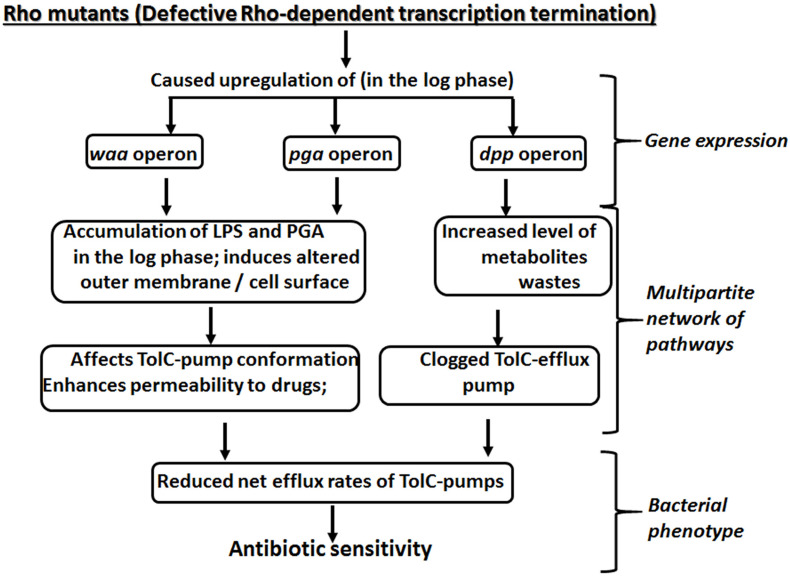
A multipartite network of pathways controlled by Rho-dependent transcription termination to yield antibiotic susceptibility.

Here we report that the compromised Rho-dependent transcription termination (mutations in the Rho protein; Chalissery et al., 2007) in *E. coli* causes reduced growth rate in the presence of a broad spectrum of antibiotics (Figure 1 and Supplementary Figure S1). Our data strongly suggest that this enhanced susceptibility to broad-spectrum antibiotics arose due to the presence of an inefficient TolC-dependent drug-efflux process in the Rho mutants (Figures 2, 3). We provided evidence that the Rho-dependent termination defects in the *waa* and *pga* operons caused accumulations of lipopolysaccharides in the OM and poly-N-acetyl-glucosamine on the cell surface during the log-phase growth (Supplementary Figure S3 and Figures 4, 5). These moieties are responsible for the sticky surface, distorted lipid bilayers, and altered cell surface textures decorated with the glycocalyx capsule of the Rho mutants (Figures 4–6). This altered cell surface architecture is strongly correlated with the antibiotic-permeability of the cell envelope (Figure 3), and reduced efficiencies of the membrane-embedded TolC-efflux pumps most likely by affecting the latter’s conformations leading to the higher net-influx rates of the antibiotics in the Rho mutants (Figures 2, 3). We also showed that the Rho-dependent transcription termination defects upregulated *dpp* operon as well as many other metabolic pathways (Supplementary Figures S5, S6 and Supplementary Table S2), enabling the mutants to utilize novel nutrients, which could be one of the major reasons for the accumulation of unusual metabolites in the Rho mutants as observed from the metabolome analyses (Supplementary Figures S8, S9). We propose that these excess metabolites in the cytoplasm of the Rho mutants could titrate the TolC-pumps, which would have further reduced their efficiencies to efflux the antibiotics. Based on the above evidence, we concluded that Rho-dependent termination is involved in the broad-spectrum antibiotic susceptibility of the Gram-negative bacteria, *E. coli*, via a multipartite network of pathways by controlling the expressions of a wide range of genes (Figure 8).

In the aforementioned section, we have proposed two reasons behind the reduced efficiencies of the TolC pumps, when Rho-dependent termination is compromised *in vivo.* However, keeping in mind the pleiotropic effects exerted by the Rho-dependent termination (Peters et al., 2012; Mitra et al., 2017), the Rho mutants are likely to have a significantly different physiology compared to their WT counterparts, which could have indirect effects on the TolC pumps via unexplored pathways other than what we described here. In this context, it is worth mentioning that we did not observe upregulation of two other well-known global regulators, csrA and crp, in the Rho mutant expressing strains (data not shown), which rules out the possibility that the observed phenotypes were indirect effects of these proteins.

Why the net influx rate of the antibiotics is faster in the Rho mutants? The upregulation of *waa* genes and *pga* operons increase the level and density of the lipopolysaccharides (LPS) and the poly-glucosamine (PGA) (Supplementary Figure S3 and Figures 4, 5; Raetz and Whitfield, 2002; Kostakioti et al., 2013) in the outer membrane of the Rho mutants during their log-phase growth. The sugar moieties of the LPS (O-antigens and the lps core) and the PGA form an intricate meshwork of carbohydrates on the surface of the cell envelope. The polar groups of the sugars could attract different antibiotics and other small molecules and deliver them to the nearby porins through a “funneling effect.” On the other hand, the high level of lipid A moieties of the LPS could also facilitate the diffusion of hydrophobic antibiotics (Tsujimoto et al., 1999). It is a well-known fact that the nature and the composition of the lipids in the membrane affect the distributions and integrity of the membrane-bound proteins, especially the transmembrane ones (Lee, 2004; von Heijne and Rees, 2008; Dowhan and Bogdanov, 2011). We speculate that the high-density presence of the lipid A moieties of the LPSs in the outer membrane is likely to influence the integrity of the transmembrane protein, TolC, and thereby could affect its efficiency of efflux. We also do not rule out the possibility that the presence of an enhanced level of the lipid and sugar moieties in the Rho mutants has a direct effect on the higher influx rate of the porins for the antibiotics.

The biofilm formed in the stationary phase of the Gram-negative bacteria acts as a barrier to antibiotic-entry, and hence bacteria is more resistant to antibiotics in their stationary phases. However, the biofilm-like structure formed in the log-phase cultures of the Rho mutants did not made the strains more resistance to the antibiotics rather they are more susceptible to it. We offer the following possible reasons for these contradictory observations. (1) The bio-film like structure in the log phase might not be similar to that observed in the stationary phase. (2) The enhanced level of LPS and sugar moieties could improve the efficiencies of the porins to transport the antibiotics as discussed in the previous paragraph. (3) Finally, we have measured the antibiotic susceptibility of the rapidly dividing log phase cultures, physiology of which is different from the antibiotic-resistant stationary phase culture having the biofilms.

The observed (Figure 1 and Supplementary Figure S1) enhanced broad-spectrum antibiotic sensitivities of the *E. coli* due to mutations in the Rho protein (N340S and G324D mutations, Chalissery et al., 2007), could have a far-reaching influence on the antibiotic treatment regimen especially against the multi-drug resistant strains. As the inhibition of the Rho function due to mutations enhances the sensitivities toward the antibiotics, the Rho-inhibitors would likely to elicit similar responses, and hence these inhibitors could be included in the co-synergistic antibiotic treatment regime together with other antibiotics, especially for curing the infections of the drug-resistant bacterial strains. A co-synergistic antibacterial effect with quinoline with the inhibition of the RecBCD by the Gam protein was earlier observed (Wilkinson et al., 2016). A study on the systematic single-gene deletions of *E. coli* strains and their susceptibility or resistivity to the Rho-inhibitor, Bicyclomycin (Tran et al., 2011), revealed that a wide range of genes is connected to Rho-dependent termination, further supports our claim for including Rho protein as a drug target in the above mentioned co-synergistic treatment regimen.

The apparent less-specificity of the Rho-loading sites on the mRNA (the *rut* sites) enables it to have a wide range of operons as well as non-operonic regions to be its targets, thereby bringing the expression of a huge number of genes under the control of this terminator. All the genomic data (Cardinale et al., 2008; Peters et al., 2009, 2012; Shashni et al., 2014) indicate that the inhibition of Rho induces expression of the whole set of new genes, which changes the physiology of the cell. Therefore, it is not surprising that Rho is involved in so many physiological processes (Mitra et al., 2017). This pleiotropic nature of the Rho function provokes us to term it as a “pleiotropic master-regulator” of the bacterial physiology. Hence, this highly conserved bacterial transcription terminator should be considered as a potential drug target, inhibition of which could block a wide range of physiological pathways simultaneously, and chances of emergence of drug-resistance against these inhibitors would be very less.

## Data Availability Statement

The raw data supporting the conclusions of this article will be made available by the authors, without undue reservation, to any qualified researcher.

## Author Contributions

MH performed the experiments and wrote the manuscript. RS designed the experiments and wrote the manuscript. Both authors contributed to the article and approved the submitted version.

## Conflict of Interest

The authors declare that the research was conducted in the absence of any commercial or financial relationships that could be construed as a potential conflict of interest.

## Footnotes

1 http://metabolomics.ucdavis.edu/

2 http://fiehnlab.ucdavis.edu/

3 https://binvestigate.fiehnlab.ucdavis.edu/#/

4 https://biolog.com/
