# Supplementary material for: The Rho-Dependent Transcription Termination Is Involved in Broad-Spectrum Antibiotic Susceptibility in Escherichia coli
Source: Front Microbiol. 2020 Nov 30;11:605305. doi: 10.3389/fmicb.2020.605305 (PMC7734253; doi:10.3389/fmicb.2020.605305)
Supplement: Supplementary file 1 [file Data_Sheet_1.pdf]

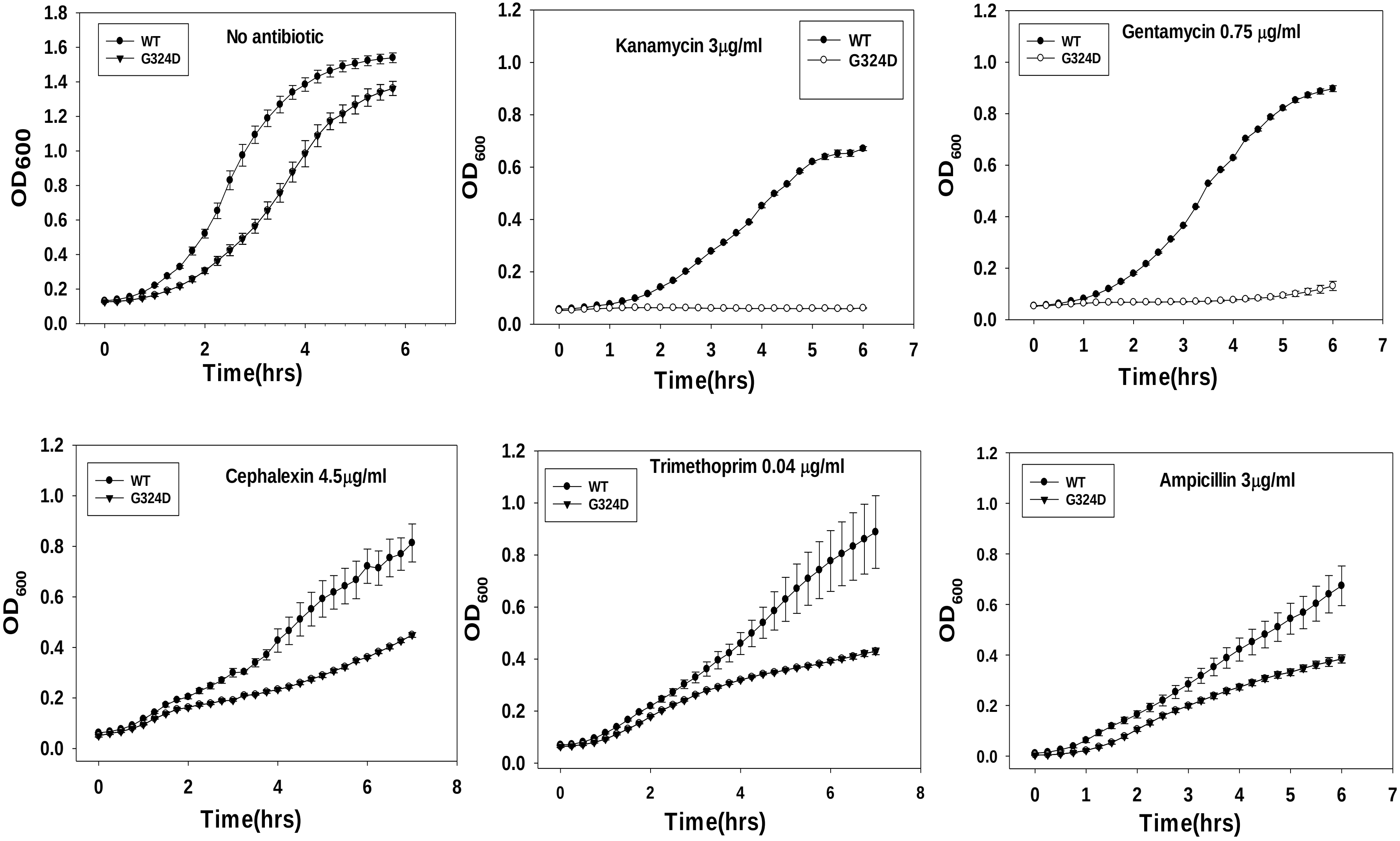

Figure S1A: MG1655 strains expressing WT or G324DS Rho proteins were grown in the presence of the indicated antibiotics in a micro titre plate. The error bars were obtained from three independent measurements.

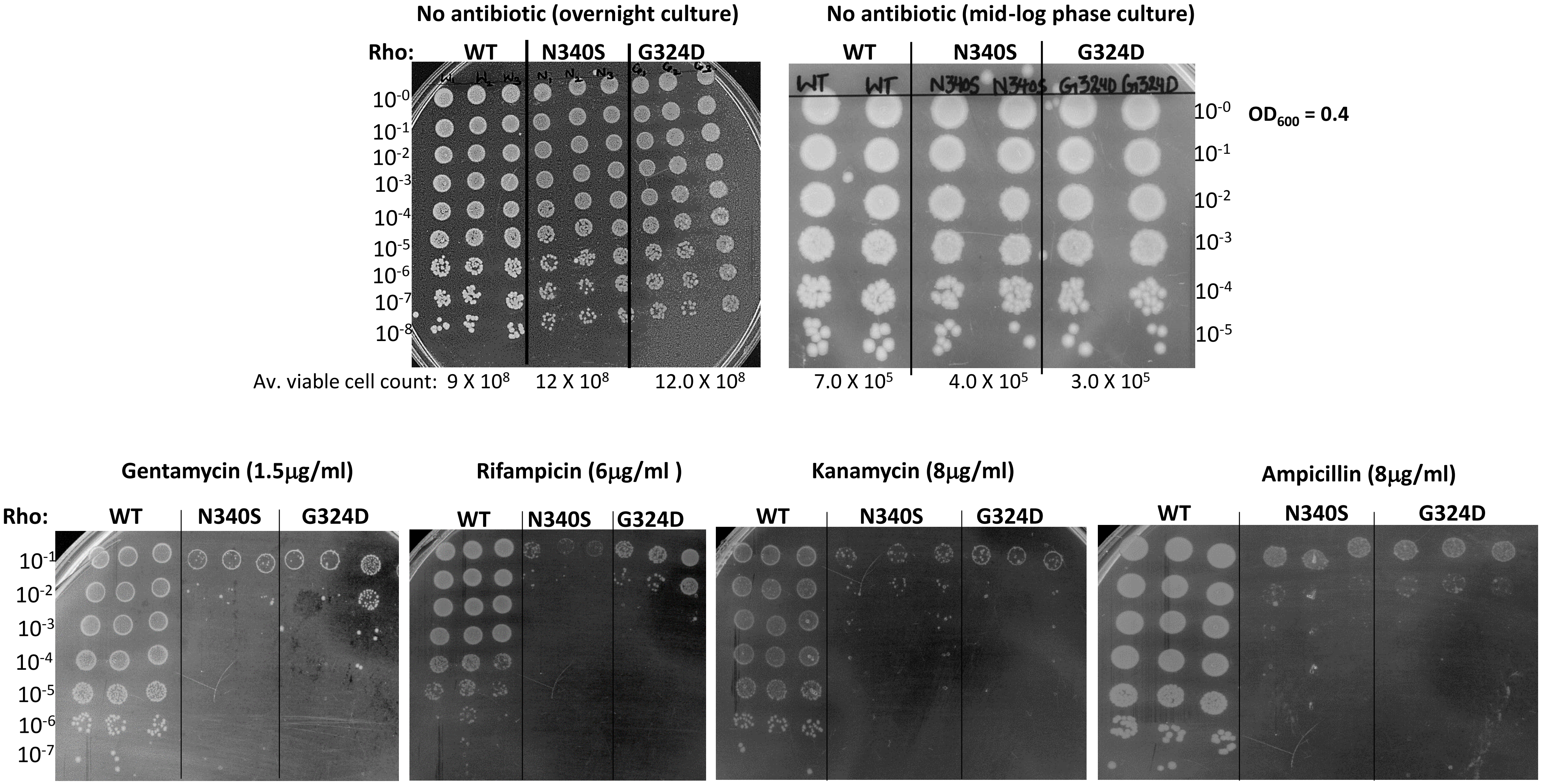

Figure S1B: Various antibiotic sensitivities of the MG1655 (RS1309) strain expressing WT and indicted Rho mutants by CFU assays. Higher sensitivities of the Rho mutants are quite evident. In each case, overnight cultures of three independent colonies were spotted on the plates containing the indicated antibiotics. Spotting assays of overnight culture and of the mid-log phase culture (~OD_{600} = 0.4) in the absence of any antibiotics is also shown as a control (top panels). Average viable cell counts are also indicated.

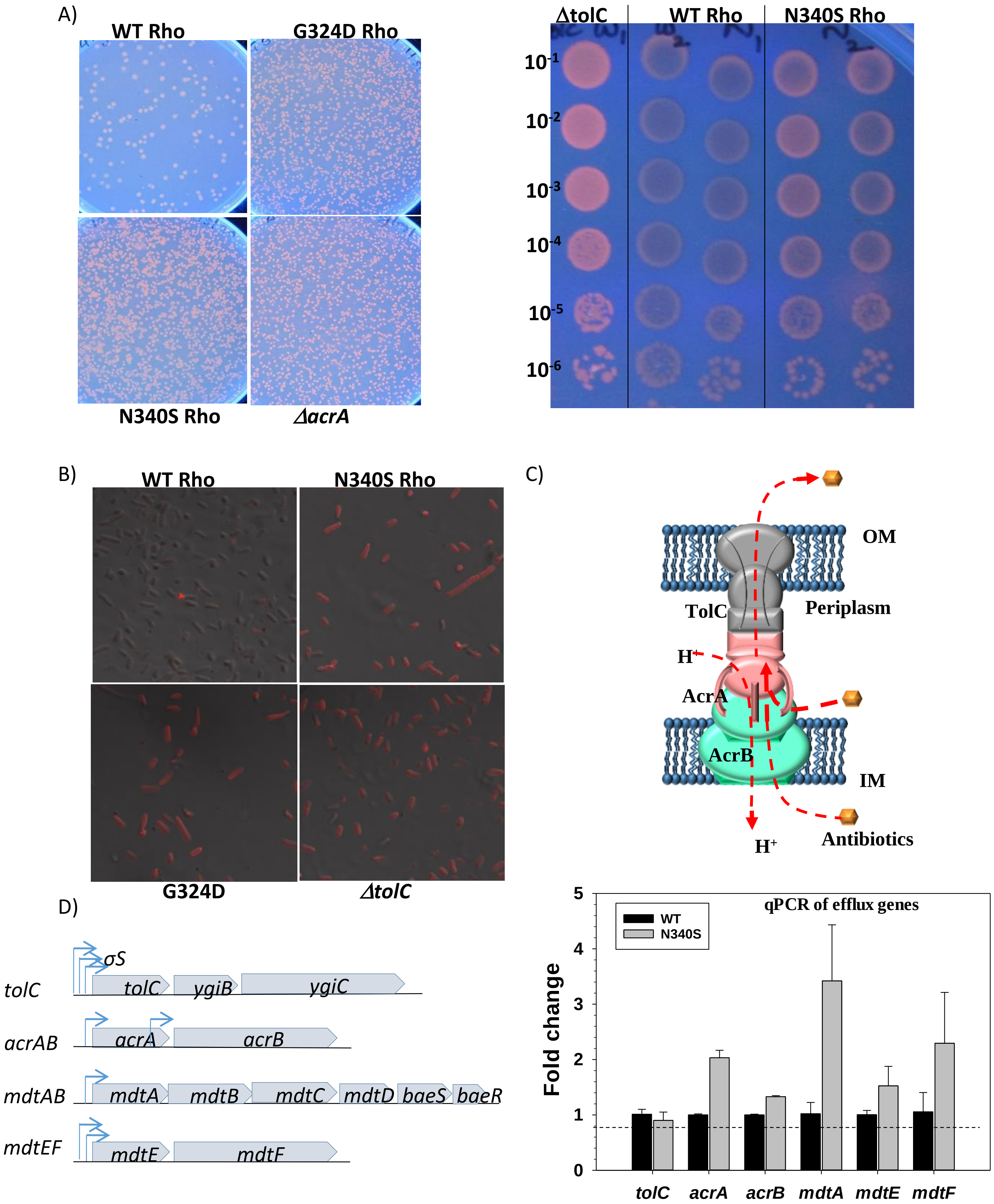

Figure S2: A) Serial dilutions of the overnight cultures of MG1655*ΔtolC*, MG1655*ΔacrA* and RS1309 expressing WT, G324D and N340S Rho proteins were spotted on LB-plates containing 0.7μg/ml EtBr (right panel), or 10^{-6} dilution was spread out onto LB-plates containing 4μg/ml EtBr (left panel).
B) Nile red uptake and staining of RS1309 and its derivatives under confocal microscope revealing the uptake of the dye of each single cell. C) Cartoons showing the organization of the TolC pump in the cell membrane (adapted from Pos KM. Proc Natl Acad Sci U S A. 2009 Apr 28;106(17):6893-4). D) Operonic arrangements of the genes corresponding to the TolC and its allied proteins (left panel) and measurements of the RNA levels of the *tolC* and its allied genes by the qPCR method (right panel). Blue arrows in left panels denote the predicted promoters in the operon obtained from EcoCyc database.

A)

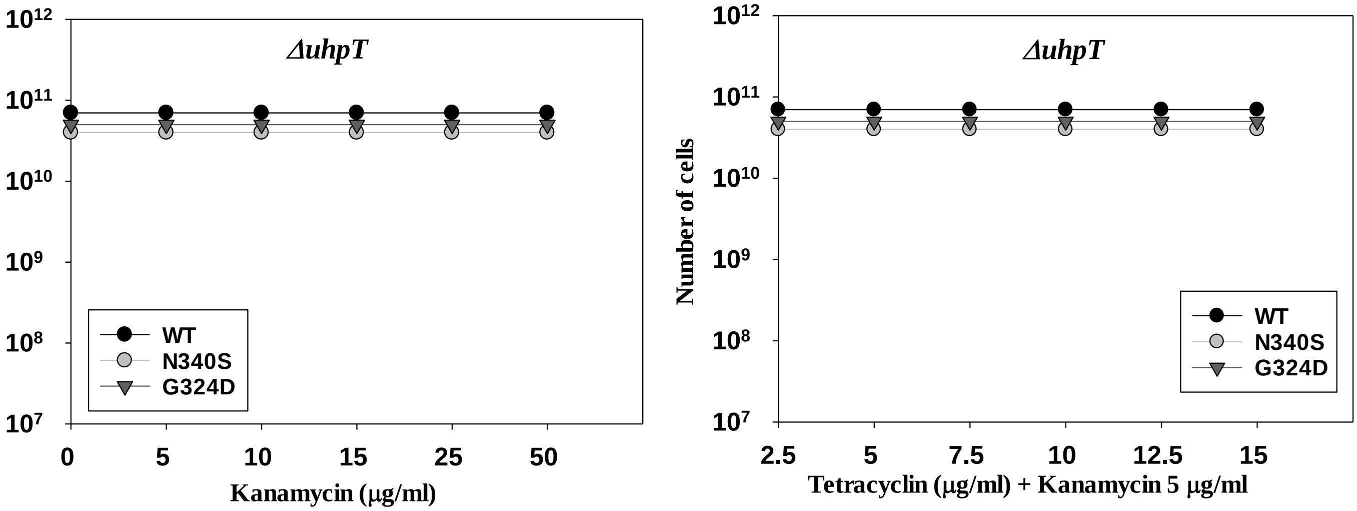

B)

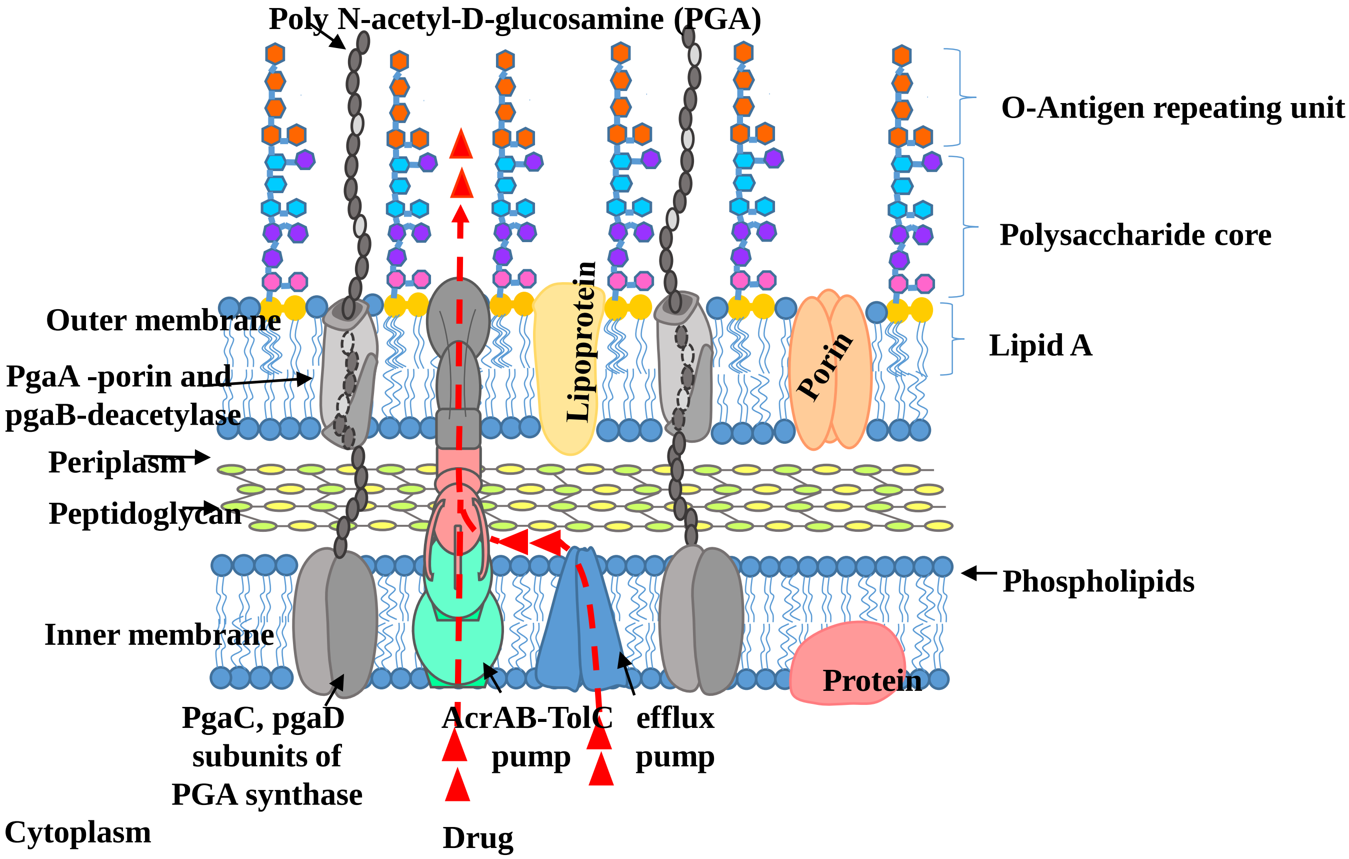

C)

Strain/ *rho*	Average OD_{490nm} from 3 measurements	Average concentrations of the hexose unit from the standard graph (mg)
WT	0.0643	0.7 ± 0.19 (1)
N340S	0.275	2.28 ± 0.22 (3.25 fold)
G324D	0.197	1.9 ± 0.19 (2.7 fold)

Figure S3: A) Number of cells survived in the presence of increasing concentrations of antibiotics as indicated upon deletion of an unrelated gene *uhpT*. B) Cartoon showing the outer membrane organization of the LPS and PGAs (adapted from Raetz and Whitefield, 2002). B) Estimate of the hexose sugar content on the cell surface of the WT and the Rho mutants. The estimation was made colorimetrically at 490nm using a standard graph obtained from known concentrations of glucose. Standard error of mean was obtained from 3 measurement.

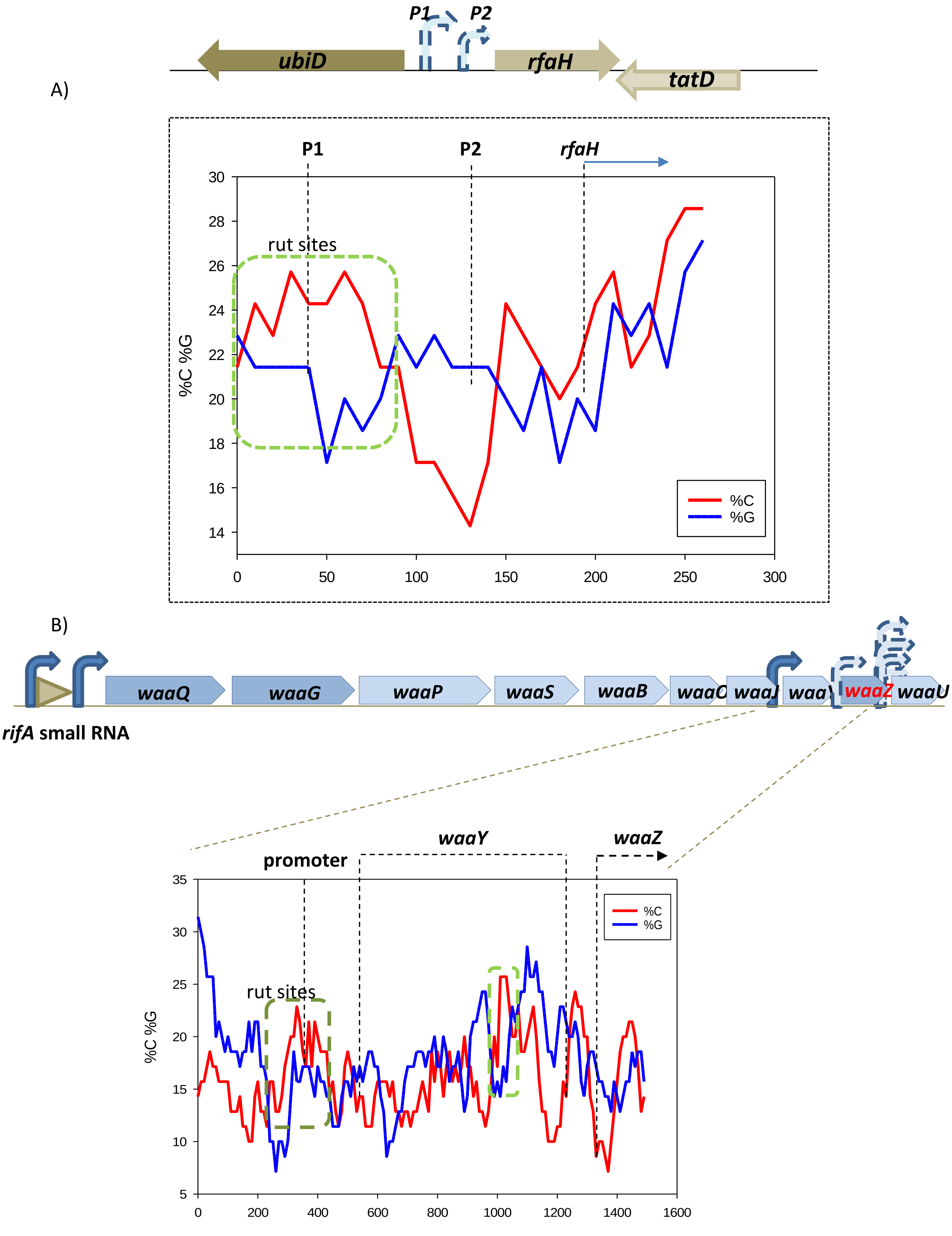

Figure S4 A, B and C: C>G plots showing the "bubble" corresponding to the probable rut sites indicated by green boxes. Predicted promoters are shown in broken arrows.

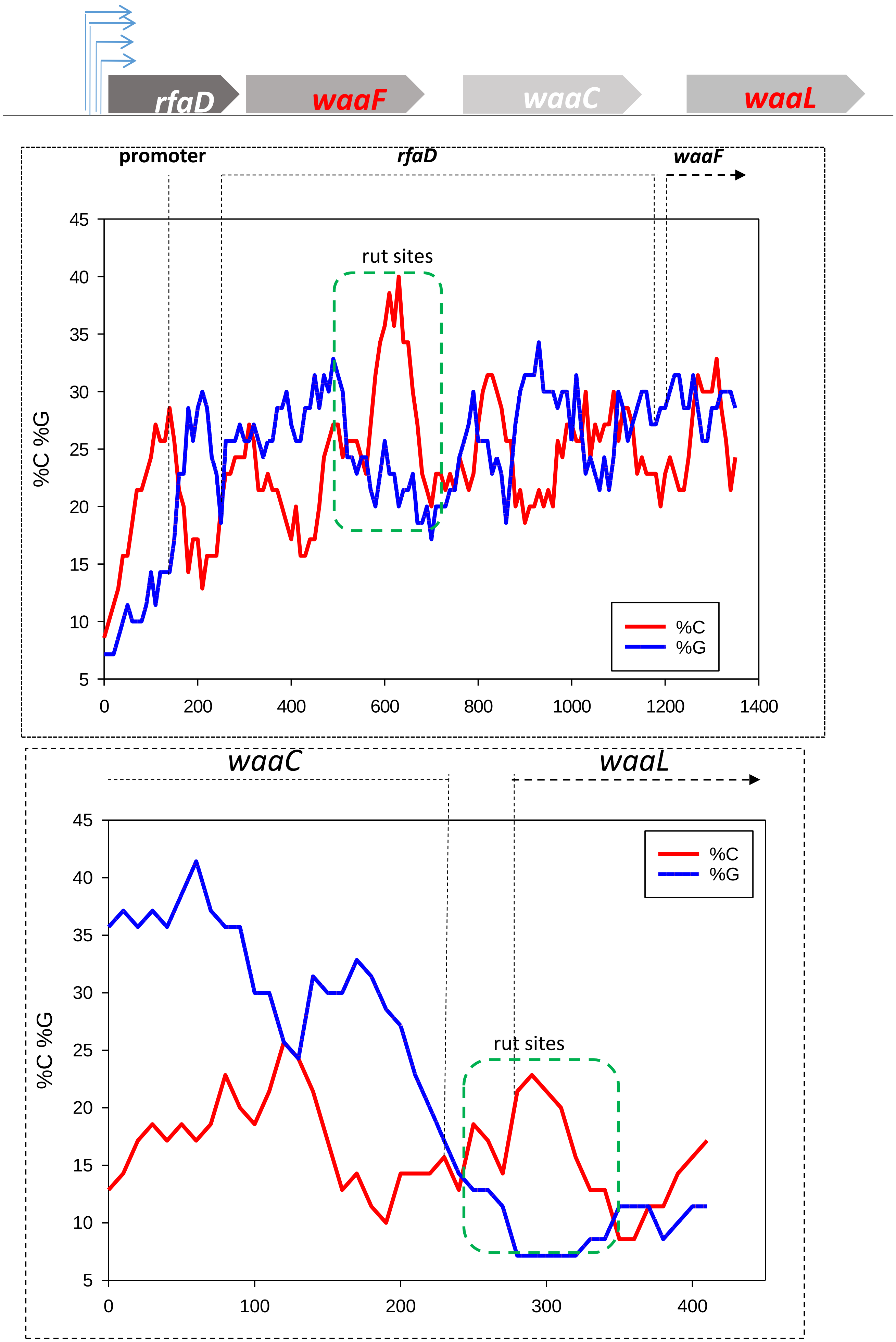
rfaD
waaF
waaC
waaL
promoter
rfaD
waaF
rut sites
%C %G
45
40
35
30
25
20
15
10
5
0
200
400
600
800
1000
1200
1400
%C
%G
waaC
waaL
rut sites
%C %G
0
100
200
300
400
%C
%G

Figure S4C

Up-regulated metabolic pathways in the Rho mutants

G324D

Pathways	Genes
Thiamine metabolism	*thiE, thiD, thiF, thiG, thiM, thiC, thiH*
2-Oxocarboxylic acid metabolism	*ilvB, ilvC, ilvN, argD, ilvE, ybhJ, ilvD, argA*
Sulfur metabolism	*amiC, cysW, cysI, cysU, cysP, cysD, cysA, cysN, cysJ*
Pantothenate and CoA biosynthesis	*ilvB, panB, ilvC, ilvN, panE, ilvE, ilvD*
ABC transporters	*proV, gltI, cysU, cysP, thiP, ugpE, fhuD, potI, cysA, livH, livG, livF, gltJ, hisP, yhdY, potH, potG, cysW, malE, malK, hisQ, ugpA, hisM, yojI, thiB, argT, afuC, gltK, yddA*
Phosphotransferase system (PTS)	*mtlA, agaD, fryA, ulaB, sgcB, sgcA, sgcC, mngA, bglF, cmtA*

N340S

Pathways	Genes
Thiamine metabolism	*thiE, thiD, thiF, thiG, thiC, thiH*
Metabolic pathways***	*puuA, carA, glcE, ddlA, gshA, thiE, luxS, hemH, metE, yjhH, ydiB, coaE, gadA, thiD, thiG, thiC, thiH, serA, eutB, purL, panB, fadB, sgcA, xapA, folB, pptA, scpA, cpsB, ilvD, puuB, sgcB, folD, uxuA, gltB, bcsZ, allB, eutA, kdgK, thiF, speC, ribA, aroH, ribC, cadA, yahI, clsA, fadE, amyA, ilvC, lysA, eutE, argD, gadB, eutD, eutG, pfkB, phoA, bcsA, ulaB, dacC, cysI, ggt, holE, purT, pgpB, gss, sgcC, gmhA, fbp*
Fructose and mannose metabolism	*mtlA, pfkB, rhaB, rhaA, fbp, rhaD, cmtA, cpsB*
Seleno compound metabolism	*ynfE, ygfM, ygfK, metE, xdhD*
Phosphotransferase system (PTS)	*mtlA, agaD, ulaB, sgcB, sgcA, sgcC, bglF, cmtA*
Taurine and hypotaurine metabolism	*gadA, gadB, ggt*

Figure S5: Upregulated metabolic pathways in the Rho mutants derived from the micro-array data of the Rho mutants (28). Genes that showed two-fold upregulation with a P-value lesser than 0.05 from microarray analysis were subjected to pathway analysis by the Functional Annotation Clustering tool in DAVID v6.7. DAVID provides enriched pathways for input genes using biological information present in the pathway databases (BBID, BIOCARTA, KEGG, PANTHER and REACTOME). The pathways with minimum gene set of 2 and cut-off p-value of 0.05 were considered significant.

<u>Nutrients utilization patterns of Rho N340S mutant strain in *w.r.t* to the WT Rho strain</u>

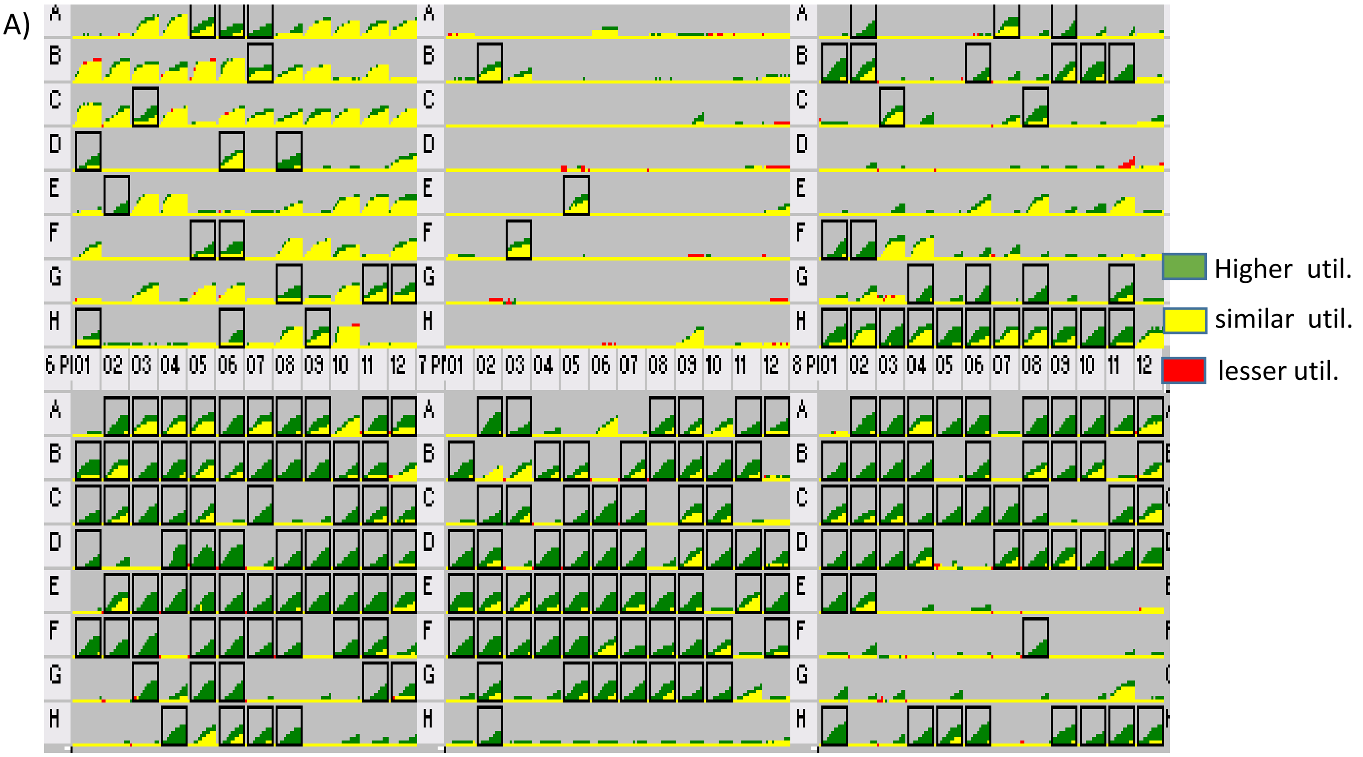

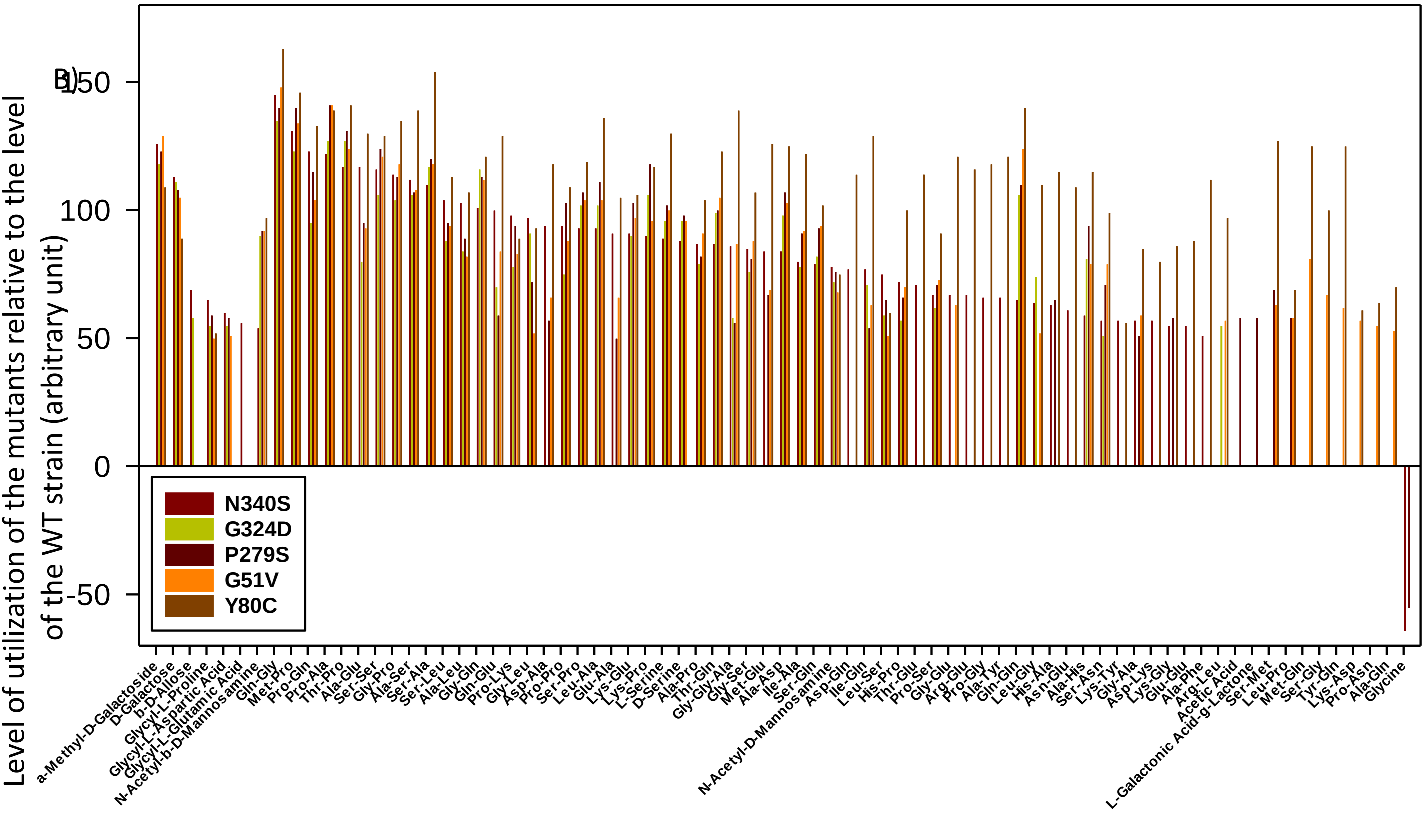

Figure S6: A) Cartoon showing the graphical representations of the Biolog plate assays in a 96-well plate well Format. Each plate has different carbon, nitrogen, phosphorous, etc sources. The area under the curve represents the utilization of particular nutrient by the mutants relative to the WT strain. Green means more utilization, Red means less utilization, while yellow means the utilization was same. The details of the nutirients are in https://www.biolog.com/wp-content/uploads/2020/05/00P134rC-PMM-Broch-PM-M1-to-M14-1.pdf. B) Level of utilizations of Nitrogen and Carbon sources of different Rho mutants. Amounts of utilizations are calculated from the area under the curves shown in figure A). The nutrient s shown here has significantly higher utilization in the Rho mutants, except for Glycine that is less utilized. Other nutrients tested were similar to the level of the WT strain.

<u>Nutrients utilization patterns of Rho N340S mutant strain in *w.r.t* to the WT Rho strain</u>

A)

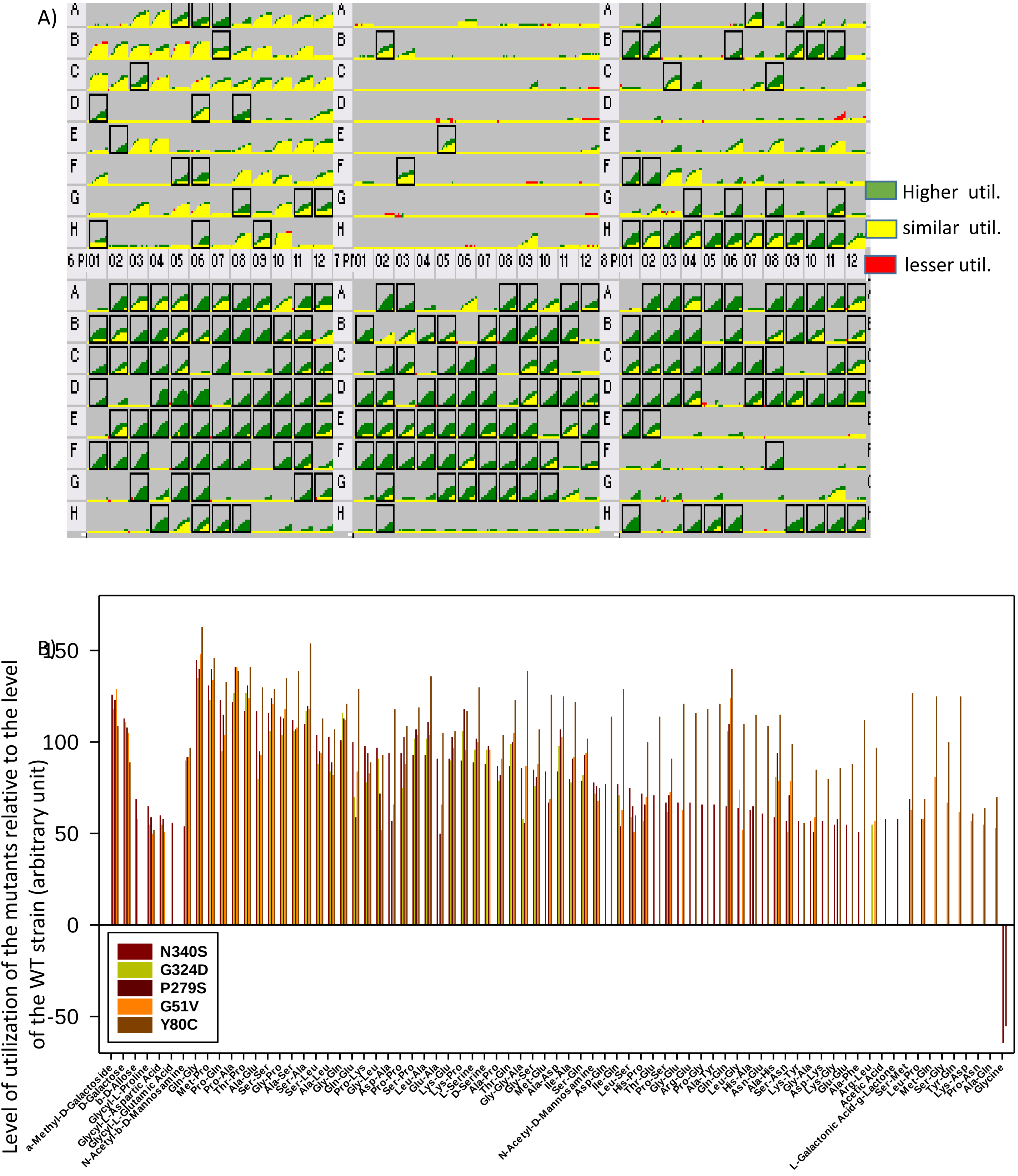

Figure S6: A) Cartoon showing the graphical representations of the Biolog plate assays in a 96-well plate well Format. Each plate has different carbon, nitrogen, phosphorous, etc sources. The area under the curve represents the utilization of particular nutrient by the mutants relative to the WT strain. Green means more utilization, Red means less utilization, while yellow means the utilization was same. The details of the nutirients are in https://www.biolog.com/wp-content/uploads/2020/05/00P134rC-PMM-Broch-PM-M1-to-M14-1.pdf. B) Level of utilizations of Nitrogen and Carbon sources of different Rho mutants. Amounts of utilizations are calculated from the area under the curves shown in figure A). The nutrient s shown here has significantly higher utilization in the Rho mutants, except for Glycine that is less utilized. Other nutrients tested were similar to the level of the WT strain.

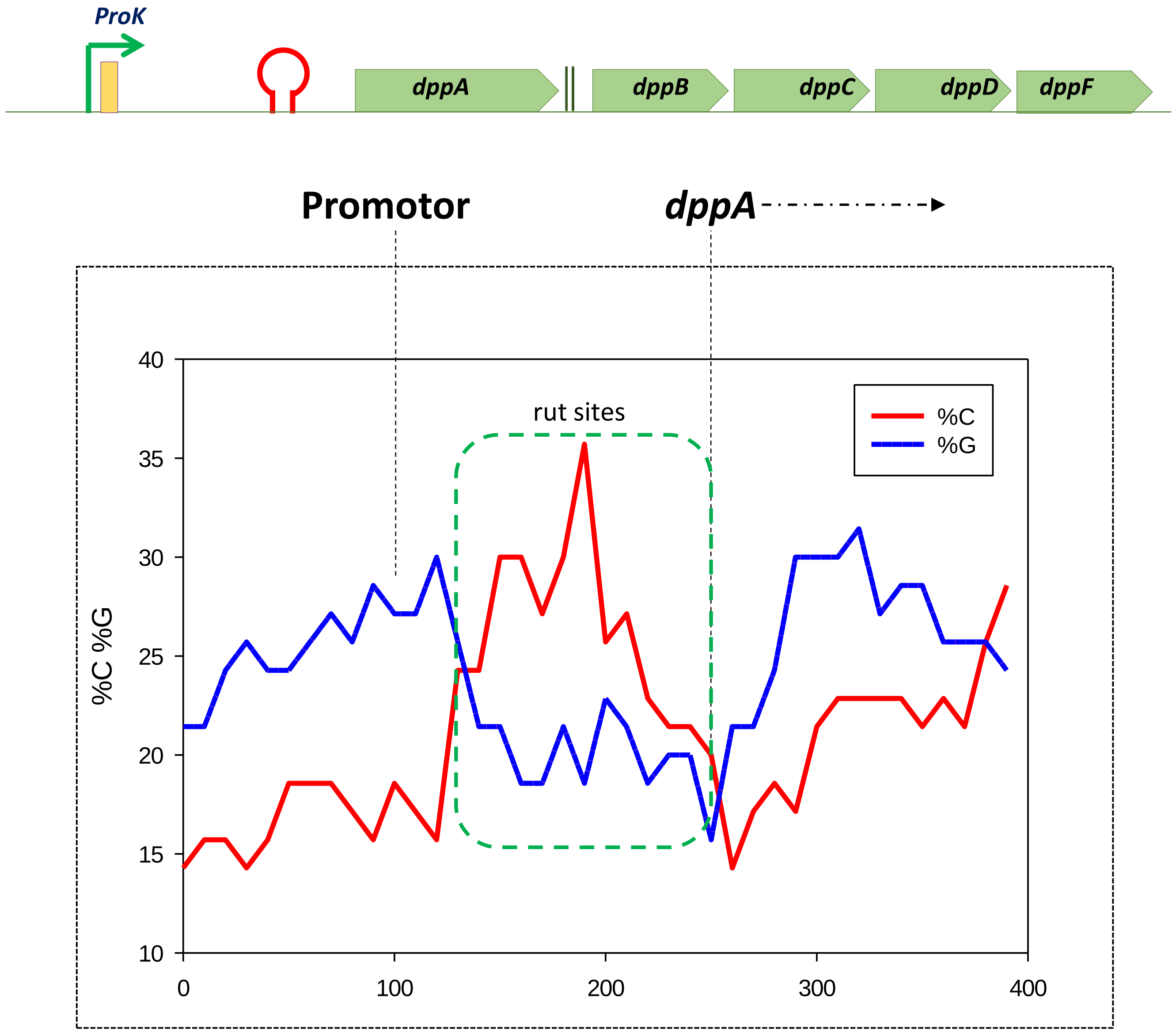

Figure S7: C>G plot showing the putative rut site upstream of the *dppA* gene indicated by a green box.

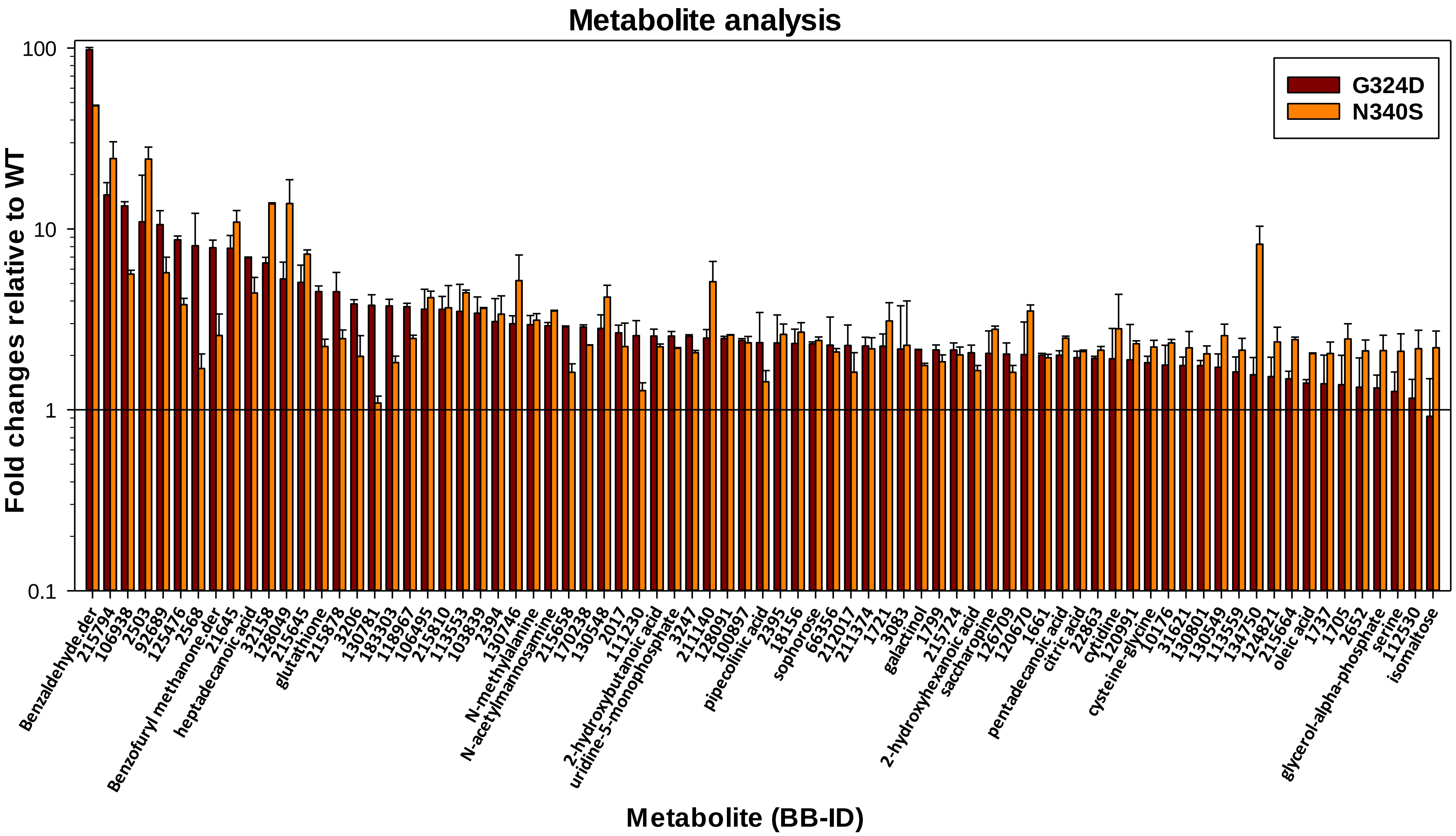

Figure S8: Primary metabolite analyses using GC-MS of the WT and the Rho mutants. The upregulated (accumulated) metabolites in the Rho mutants relative to that of the WT strains are shown as fold changes. Unknown metabolites are expressed as the binbase id numbers. The highest upregulation was (97 fold upregulation in G324D and 47 fold upregulation in N340S) observed for a benzaldehyde derivative (binbase id-215855). Errors were calculated from two biological replicates.

Details of the Metabolites from the GC-MS analyses

Description	G324D	N340S
Total number of metabolites	703	703
Known metabolites	185	185
Unknown metabolites	518	518
Maximum Upregulation	~98-fold	48 fold
Maximum Downregulation	20.3 fold	8.5 fold
>10 fold Upregulation	5	6
>10 fold Downregulation	2	
>2fold Upregulation	64	71
>2 fold Downregulation	40	39
Between 1-2 fold upregulated	401	384
Between 1-2 fold downregulated	238	248

Figure S9: Level of metabolites in the Rho mutant strains, N340S and G324D expressed in fold change relative to those obtained in the WT strain.
