# Supplementary material for: The Rho-Dependent Transcription Termination Is Involved in Broad-Spectrum Antibiotic Susceptibility in Escherichia coli
Source: Front Microbiol. 2020 Nov 30;11:605305. doi: 10.3389/fmicb.2020.605305 (PMC7734253; doi:10.3389/fmicb.2020.605305)
Supplement: Supplementary file 2 [file Table_1.DOCX]

**Table S1: Estimate of IC_50_ (50% survival) of each of the antibiotics.**

| **Antibiotics** | **MG1655 WT *rho*** | **MG1655 N340S r*ho*** | **MG1655 G324D r*ho*** |
| --- | --- | --- | --- |
| Kanamycin | ~7 μg/ml | ~3.5 μg/ml | ~4.0 μg/ml |
| Trimethoprim | ~0.4 μg/ml | ~0.03μg/ml | ~0.025μg/ml |
| Ampicillin | ~5 μg/ml | ~3 μg/ml | ~4.0 μg/ml |
| Nalidixic acid | ~3 μg/ml | ~2.5 μg/ml |  |
| Erythromicin | ~20 μg/ml | ~8 μg/ml |  |
| Rifampicin | ~60 μg/ml | ~7.5 μg/ml | ~4.0 μg/ml |
| Chloramphenicol | ~1 μg/ml | ~0.5 μg/ml |  |
| Gentamycin | ~1.4 μg/ml | ~0.7 μg/ml | ~0.75 μg/ml |
| Chephalexin | ~6.0 μg/ml | ~4.25 μg/ml | ~3.75 μg/ml |

Indicated strains were grown in the presence of varying concentrations of antibiotics. Approximate concentrations of each of the antibiotics that caused a 50% reduction in growth @ 37^o^C in the LB media are tabulated. These values are obtained either from the spotting assays or from the stationary phases of the growth curves.
