# Supplementary material for: The Rho-Dependent Transcription Termination Is Involved in Broad-Spectrum Antibiotic Susceptibility in Escherichia coli
Source: Front Microbiol. 2020 Nov 30;11:605305. doi: 10.3389/fmicb.2020.605305 (PMC7734253; doi:10.3389/fmicb.2020.605305)
Supplement: Supplementary file 4 [file Table_3.docx]

| **Table S3 Primers:** |  |
| --- | --- |
| RS1308 | AGCAGCTGCGCCAGATCACC-FP for *tolC* middle region for RT-PCR |
| RS1309 | CAGAAATCCCGGTAGAAGCCG-RP for *tolC* middle region for RT-PCR |
| RS1312 | ACACCAAAGTCACCTCTCCG- FP for *acrA* middle region for RT-PCR |
| RS1313 | GCCGTTCTCTTGTTTCAGCG- RP for *acrA* middle region for RT-PCR |
| RS1314 | CCATGCTGAAACCGATTGCC- FP for *acrB* middle region for RT-PCR |
| RS1315 | CAGACGCACGAACAGATAGG- RP for *acrB* middle region for RT-PCR |
| RS1546 | GGATCTCTTTTACAGCCACC-FP for *pgaA* middle region for RT-PCR |
| RS1547 | GTCATTTCAGCCTGAGGAAG-RP for *pgaA* middle region for RT-PCR |
| RS1548 | AACCTCCAGCAAATGGATCG-FP for *pgaB* middle region for RT-PCR |
| RS1549 | GATGTTTACACCTGAGCGGG-RP for *pgaB* middle region for RT-PCR |
| RS1550 | CCTGGTGGGTAAAATTCAGG-FP for *pgaC* middle region for RT-PCR |
| RS1551 | CTGATTCAACTGCAGCTTCC-RP for *pgaC* middle region for RT-PCR |
| RS1552 | CTTATTCGCCATGGATCTGC-FP for *pgaD* middle region for RT-PCR |
| RS1553 | GGTATTGCTAAGCTCTCTGC-RP for *pgaD* middle region for RT-PCR |
| RS1371 | GACTGGAGCCGGATTACCG- FP for *mdtA* middle region for RT-PCR |
| RS1372 | GATCCCAGGCTTCTACCACC- RP for *mdtA* middle region for RT-PCR |
| RS1373 | ACAGGCGACGATCAATCTGC- FP for *mdtE* middle region for RT-PCR |
| RS1374 | GCGACCTCTTCTTTCATGCG- RP for *mdtE* middle region for RT-PCR |
| RS1375 | CAAACCTAACGCCCTGTTCG- FP for *mdtF* middle region for RT-PCR |
| RS1376 | TACCCCCTGATCCTCTTCTGG- RP for *mdtF* middle region for RT-PCR |
| RS1466 | TTTGATGGCTACTGGGGCAC- FP for *dppA* middle region for RT-PCR |
| RS1467 | AGATAACCGACGTTCAGCCC- RP for *dppA* middle region for RT-PCR |
| RS1468 | TTCGATCACACAGCGGTTGG- FP for *dppB* middle region for RT-PCR |
| RS1469 | AGATGGCGGTGTCGATTAGC- RP for *dppB* middle region for RT-PCR |
| RS1470 | CACTGACCTTCGTTGCCTTG- FP for *dppC* middle region for RT-PCR |
| RS1471 | TGGCGTTAGAGAAACCGAGC- RP for *dppC* middle region for RT-PCR |
| RS1472 | CGTCTGGATGTTTACCCGC- FP for *dppD* middle region for RT-PCR |
| RS1473 | GCGCCATGTTCTCTTTCTGC- RP for *dppD* middle region for RT-PCR |
| RS1474 | CACTATGACCGCTATCCGC- FP for *dppF* middle region for RT-PCR |
| RS1475 | TAAGACAGCCCCAACTCCTG- RP for *dppF* middle region for RT-PCR |
